# Learning analytics for enhanced professional capital development: a systematic review

**DOI:** 10.3389/fpsyg.2024.1302658

**Published:** 2024-01-22

**Authors:** Javier de La Hoz-Ruiz, Mohammad Khalil, Jesús Domingo Segovia, Qinyi Liu

**Affiliations:** ^1^Department of Didactics and School Organization, University of Granada, Granada, Spain; ^2^Center for the Science of Learning & Technology, Faculty of Psychology, University of Bergen, Bergen, Norway

**Keywords:** professional capital, learning analytics, social capital, professional learning community, communities of practice

## Abstract

**Background/Motivation:**

This article presents a systematic review aimed at examining the utilization of learning analytics (LA) to enhance teachers’ professional capital.

**Aim:**

The study focuses on three primary research questions: (1) exploring the characteristics and approaches of LA in professional capital, (2) investigating suggestions from LA for assessing and improving professional capital, and (3) examining variables studied in enhancing the most intricate dimension of professional capital using LA.

**Methodology:**

To address the research objectives, a systematic review was conducted focusing on the key concepts “learning analytics” and “professional capital.” Following the procedures outlined encompassed in four stages: identification, screening, inclusion, and adequacy. The PRISMA 2009 protocol guided the systematic review process.

**Principal findings:**

The findings of the study underscore the efficacy of LA as a catalyst for improving professional capital, particularly through collaborative learning and the utilization of tools like forums and online learning platforms. Social capital emerges as a pivotal component in integrating diverse types of professional capital, fostering opportunities for knowledge creation and social networking.

**Conclusion/Significance:**

In conclusion, the study highlights the paramount significance of addressing teachers’ professional capital development through collaborative approaches and leveraging technology, particularly in primary education. The article concludes by emphasizing the imperative for more research and knowledge dissemination in this field, aiming to ensure equity in learning and address the challenges posed by the COVID−19 pandemic.

## 1 Introduction

Achieving goals 4 and 17 of the 2030 Agenda on Sustainable Development Goals (SDGs) is currently a major challenge for education systems and requires the collaborative efforts of teachers, families, communities and students. Goal 4 aims to ensure inclusive and equitable quality education, promoting learning opportunities for all. On the other hand, Goal 17 focuses on strengthening the implementation of SDGs through global partnerships. In this context, addressing educational challenges (Goal 4) involves the collaboration of teachers, families, communities, and students. This collaboration is essential to ensure equitable access to quality education and contribute to sustainable development. Key indicators include the educational completion rate, access to preschool, the proportion of trained teachers, foreign investments, and international cooperation. The interconnection of these goals highlights the need for coordinated efforts to make a meaningful impact on education and sustainable development.

In 2021, UNESCO highlighted the pressing need for new schools and governance structures with the capacity to energize and unify a community of individuals with the shared purpose of seeking knowledge while being committed to enhancing the quality and equity of education. In this regard, the action of school leaders and the advancement of these schools and communities as extended communities of professional practice are fundamental (Bolívar and Domingo, 2023). The concept of Third Generation Professional Learning Communities is central to this discussion. These communities — in addition to their core goal of improving the learning of all and among all — aim to foster internal capacities for improvement through interactive and committed professionalism (Hargreaves and O’Connor, 2018). This approach also places a significant emphasis on accumulating more professional capital (Hargreaves and Fullan, 2012, 2013, 2020) generated through the extension of the community of practice into to the school environment, local community, and across professional and inter-institutional networks. As a result, there is pressing needs for fluid networks of interrelation, communication, and support for learning for all and for all, with a shared and networked form of leadership, unified by a broad perspective of “leadership from the middle” (Rincón, 2019).

These new scenarios outlined by UNESCO in 2021, coupled with the complexity of the current post-pandemic era and the transition toward a “new normal” (Hargreaves, 2020; Darling-Hammond, 2022), highlight the need for educational institutions to adapt. This adaptation is particularly crucial in challenging and vulnerable contexts (e.g., communities with limited resources, schools with access challenges). In turn, there is a growing need for more innovative and engaged schools, which involves embracing new forms of governance that foster their development as communities of professional practice. Such communities’ initiate projects and create cultures and environments with a shared commitment to educational improvement. Thus, the educational improvement largely depends on the ability of school leaders to connect everyone (teachers, families, and the local community) to form Communities of Professional Practice (Kimble and Hildreth, 2008). Professional capital plays a fundamental role in the building these internal capacities for improvement (Hargreaves and Fullan, 2012). This concept is one of the central issues of the present work, together with the sense of sustainability and quality of education.

Education systems are at a crossroads in their efforts to facilitate professional learning for teachers that supports both institutional improvement and ensures quality and equity of learning and sustainable development. Therefore, it is of great interest to the academic field to identify the measures that are being used to extract information about professional capital as well as to investigate the improvement processes. However, few studies have focused on informing the design and implementation of intentional frameworks to enhance teachers’ professional capital through the growth of social networks (Yoon et al., 2018). To fill this gap, it is worth highlighting the potential of the emerging field of *learning analytics (hereinafter LA)*.

LA aims to develop tools to raise awareness of the presence of learning activities and processes, i.e., to make such processes available for analysis. LA can be applied to collect and analyze information about teaching activities and can also help to improve professional capital. A review of previous studies on professional capital reveals the existence of sufficient knowledge regarding the utilization of learning analytics for developing skills related to this capital (Tong and Razniak, 2017; Jan et al., 2019; Silva et al., 2020; Demir, 2021; Minga-Vallejo et al., 2021; Yassine et al., 2022). However, to the best of our knowledge, there are no systematic reviews focusing on the application of LA to improving professional capital. While previous works by Tong and Razniak (2017) and Demir (2021) explored aspects of professional capital, they focused on collaborative leadership and social capital, respectively, without specifically considering LA as a means of improvement. Similarly, Jan et al. (2019), Silva et al. (2020), Yassine et al. (2022), and Minga-Vallejo et al. (2021) covered to a large extent Social Network Analysis (SNA) and online learning environments but did not concentrate on professional capital improvement through LA, to name but a few. Despite providing valuable insights, these studies leave a gap in understanding how LA can be specifically employed to enhance professional capital. Recognizing this gap is crucial for guiding future research in this area.

Gaining insight into the most recent studies in this field holds great significance for researchers seeking to uncover potential avenues for future exploration in learning analytics, ultimately contributing to the advancement of professional capital. To that end, we follow a systematic literature review to answer the following research questions:

*RQ1*: What are the characteristics and approaches of existing learning analytics in professional capital?

*RQ2*: What does learning analytics suggest should be evaluated and analyzed to improve professional capital?

*RQ3*: What is the most untangled dimension of learning analytics in professional capital, and how learning analytics is improving this dimension?

The review study is structured as follows: we begin by establishing a pertinent background, followed by a narrative that reports on our systematic review of the literature. We then presents the results of our synthesis and discuss significant insights and findings. Lastly, we discuss limitations and draw conclusions.

## 2 Professional capital: human, social, and decisional capital

Professional capital refers to the skills and knowledge that a person possesses and that allow them to perform their work effectively, as well as the relationships and networks they build within the educational environment. Professional capital — the most important factor in social production and activity — is a concept related to the value of individuals or groups and can be used to enhance long-term growth (Giddens, 1999).

In the educational field, this term refers to the combination of knowledge, skills, and experience that an education professional possesses and that are valuable to their work performance in education. Day et al. (2006) define professional capital as theoretical and practical knowledge acquired through initial and continuing training, as well as professional experience in the educational field, all of which enable the teacher to develop effective pedagogical and didactic skills and competencies. On the other hand, Hargreaves et al. (2002) define it as knowledge, skills and competences acquired through critical reflection and experiential learning, which enable the education professional to make informed decisions and develop effective strategies to improve teaching and learning. Organization for Economic Co-operation and Development (2019) considers this term as the combination of knowledge, skills, competencies, and values that an education professional possesses, and that allows them to exercise their work ethically and effectively in the educational field.

It is worth noting that the world’s highest-performing education and economic systems are adopting the strategy of fostering professional capital. Countries and communities investing in professional capital are therefore making a long-term investment in developing human capital.

The key to this concept is systemic development and the integration of three types of capital – human, social, and decisional – into the teaching profession. Professional capital is concerned with collective responsibility (rather than individual autonomy), rigorous training, continuous learning, going beyond the evidence, and being open to the needs and priorities of students and society (Hargreaves and Fullan, 2012).

From this standpoint, developing good teachers for all students requires teachers to be highly committed, well prepared, engaged in continuous training, adequately paid, and involved in good teamwork to maximize their own progress and make effective judgments by using all their ability and experience (Hargreaves and O’Connor, 2018).

From an economic perspective, the process of teacher professional development is a worthwhile, long-term investment that will add to the net value of professional capital. By investing in innovative, professional, and high-quality teachers, their professional capital can be increased and circulated, and the teachers will be expected to introduce significant innovations into their teaching practice (Liu et al., 2020). To implement innovative teaching in a challenging educational environment, teachers must have high levels of professional capital and make appropriate investments in professional practice to improve performance.

According to Tong and Razniak (2017), the development of effective professional capital requires collaborative leadership, professional development, and adult learning. As the collaborative culture gains momentum within the school environment, greater collaboration promotes inclusion, trust, risk-taking, and fosters connectivity among staff. According to DuFour (2003), capacity building through collaborative teamwork is important for cultivating a positive learning environment for all. One of the important factors that administrators should consider is being aware of the challenges within their school community. In other words, teacher engagement can begin to generate professional capital (Hargreaves and Fullan, 2012), i.e., teaching wisdom, collaborative ability, and mastery of the content they develop, which can extend from the cloud to the classroom (Hu et al., 2018).

To view the entire process of school education from the perspective of professional capital, it is important to highlight the three dimensions indicated by Hargreaves and Fullan (2012): (a) human, (b) decision-making and (c) social capital.

### 2.1 Human capital

Human capital within the field of education refers to the set of skills, knowledge, experiences, and competencies that people possess and that allow them to exercise their educational work effectively. Importantly, human capital in education refers not only to the skills and knowledge of education professionals, but also to the skills and competencies of students that are the result of the education and training they receive (Coleman, 1988).

Some of the most notable definitions of human capital within the educational area include that of Schultz (1961), who defines this construct as the set of knowledge, skills, and values that individuals possess and that have been acquired through education, training and experience, which allow them to perform effectively in the educational field. For Becker (2010), human capital is the set of competencies and skills that enable individuals to perform effectively in the workplace, including the ability to adapt to changing situations and learn continuously. In the case of education professionals, this refers to their ability to enhance student learning.

### 2.2 Decision-making capital

The term “decisional capital” refers to the power that a person or entity has to make important and strategic decisions in an organization, company, or institution. Some important definitions related to this concept are presented by authors such as Simon (1977), and Grant (1996), by which they highlight the influence of a person, group, or entity when it comes to making important decisions that impact the functioning of an organization. In business terms, Nonaka and Takeuchi (1995) conceive this power as the ability of a person or entity to make decisions and carry out actions that significantly determine the success or failure of a company or project in the short and long term.

The significance of decisional capital within the broader framework of professional capital has been addressed in the literature. For example, McKenzie et al. (2011) assert that improving the governance of the decision-making process will yield progressive benefits through the meticulous planning of developmental initiatives. In addition, Visone (2018) shows how leaders who trust teachers, value their contributions, and provide opportunities for decision-making and leadership also supported the development of decisional capital and social capital, a concept that will be the focus of the next section.

### 2.3 Social capital

In education, social capital refers to the benefits that individuals may derive from their connections and relationships with others (Coleman, 1988) by allowing them access to assets such as information, advice, experience, materials, and confidence that can facilitate positive changes in teachers’ beliefs and practices (e.g., Daly et al., 2010; Penuel et al., 2013). As Leana (2011) states, high social capital generates an increase in human capital. Thus, for example, if efforts are focused on increasing individual talent, much more work will be required to build social capital. In contrast, individuals gain confidence, learn, and receive feedback by being surrounded by the right types of people and having the appropriate relationships and interactions in their environment.

A growing body of work has sought to identify the mechanisms that facilitate social relations among teachers, pointing to various characteristics of educational infrastructure that support social capital as a source of development in schools, such as grade level assignment and formal leadership positions (Spillane et al., 2015). However, it is important to recognize that normative dimensions play a pivotal role in fostering social relationships. In schools where teachers adhere to shared norms such as trust and collective responsibility, in schools where teachers adhere to shared norms such as trust and collective responsibility, there is a greater likelihood of improvement (Bryk and Schneider, 2002).

### 2.4 Learning analytics and professional capital

Learning analytics is defined as the measurement, collection, analysis and presentation of data on students and their contexts, in order to understand and optimize learning and the environments in which it occurs (Long and Siemens, 2011). LA has considerable value because it can be used as a means to extract methodologies and more effective processes and tools in data measurement, collection, analysis, and reporting of professional capital (Khalil and Ebner, 2016). According to Khalil and Ebner (2016), the methods of LA can be categorized as follows: (a) data mining techniques; (b) statistics and mathematics; (c) text mining, semantics, and linguistic analysis; (d) display; (e) social network analysis; (f) qualitative analysis; and (g) gamification.

There are several discussions including institutional reports such as D2L’s “The State of Learning Analytics in 2020” (2020) demonstrating the importance of the field in education. This report brings insight on the increasing usage of data to personalize education and improve the student experience. The field is also strongly related to Artificial Intelligence (AI) where LA depends on methods of AI to understand learning; for example, machine learning and related data-driven approaches. For that reason, it should be noted that LA is a combination of different disciplines such as computer science, statistics, psychology, and education.

In general terms, learning analytics allows us to identify opportunities for improvement in the teaching and learning process. This can help professionals identify the skills and knowledge they need to improve their performance and therefore their professional capital. In addition, LA can help generate professional capital by improving the teaching and learning process, enhancing the retention and completion of training programs, and identifying trends and patterns in the workforce.

Having explained the key variables, the present study proposes how these should be worked on by identifying the lines of research and their interconnections based on the information contained in the databases via carrying out a systematic literature review to understand the structure and knowledge gaps of the scientific domain.

## 3 Methodology

In order to respond to the research objectives, a systematic review was conducted, focusing on the two key concepts to be analyzed: “learning analytics” and “professional capital.” We adopted the procedures of Kitchenham and Charters (2007) and Pollock and Berge (2018) when carrying out the systematic review, which comprises four stages: identification, screening, inclusion, and adequacy. The PRISMA 2009 protocol was also adopted as a guide for producing this systematic review (Liberati et al., 2009).

### 3.1 Identification and screening

#### 3.1.1 Identification: data source

The following databases and their rational were utilized in this study:

1. Web of Science (WOS) is one of the most reputable collections of journal articles, indexing both the Social Science Citation Index (SSCI) and Science Citation Index (SCI).^1^
2. Scopus is a database of great international relevance. Like the SCI, it not only collects bibliographic information, but also analyzes the behavior of the citations received by journals, which allows generating a large number of bibliometric and citation indicators, such as the h index.
3. ACM is the database chosen for the topic of this study, being “the most complete full-text database in the world for articles and bibliographic literature on computing and information technology.” The annual proceedings of the Learning Analytics and Knowledge Conference (LAK) are published in the ACM Digital Library.

#### 3.1.2 Search and screening strategy

**Figure 1:**
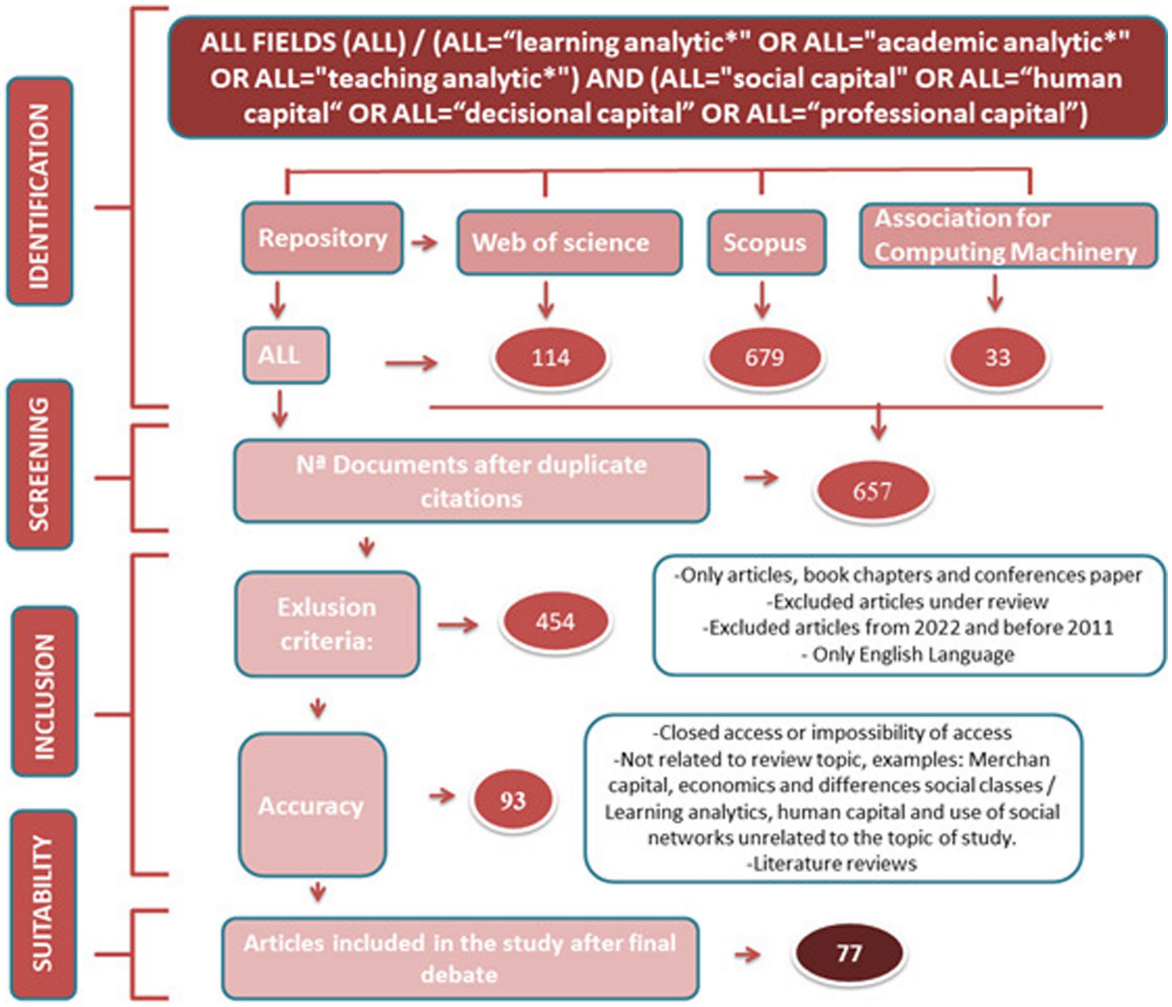
Search and selection process of studies to be reviewed.

The following key terms were integrated in the systematic review and used in the search formula: ALL FIELDS/ (ALL = “learning analytic*” OR ALL = “academic analytic*” OR ALL = “teaching analytic*”) AND (ALL = “social capital” OR ALL = “human capital” OR ALL = “decisional capital” OR ALL = “professional capital”) in the three databases, after duplicate citations, 657 articles were extracted at this initial stage. The words used in the search equation are the general terms related to the focus of the study (learning analytics, professional capital) accompanied by their sister terms within the area (academic analytics, teaching analytics, social capital, human capital, decisional capital). We decided to include “learning analytics,” “academic analytics,” and “teaching analytics” to expand our search umbrella since various authors have debated conceptual differences between the three terms, but common practice often employs them interchangeably. By incorporating the three, we aim to encompass a broader spectrum of research and relevant resources. The search was conducted on February 23, 2022, after which the articles were subjected to the process of reading, screening, and analysis. Figure 1 shows the search and selection process for the studies reviewed.

### 3.2 Inclusion and eligibility criteria

After specifying the above search terms, the search was further narrowed by applying the following criteria as described in Table 1, resulting in a final corpus of 77 articles.

**Table 1:** Inclusion and exclusion criteria of the systematic review.

Criteria	Inclusion	Exclusion
Topic and focus of study	Use of Learning analytics to improve professional capital in education.	Mercantile capital, economy, and different social classes. Learning analytics, human capital, and use of social networks not related to the subject of study.
Language	English	Other language
Publication period	January 2011–December 2021	Articles excluded 2022 onward and those before 2011 (the field of learning analytics emerged in 2011)
Type of publication	Articles, book chapters, and conference communications	Books, posters, workshop documents, editorials, and reports.
Publication status	Peer-reviewed articles	Non-peer-reviewed and in-press articles
Other	Accessible	Inaccessible, and literature reviews

We also adopted a quality assessment as referred by Schön et al. (2017). Table 2 displays the checklist used to assess the quality of the included studies. All primary studies (77 documents) were evaluated on the basis of quality indicators.

**Table 2:** Quality criteria used to assess the adequacy of the study.

Item	Assessment criteria	Score	Description
QA1	Were the objectives of the research clearly stated?	−1	The objectives were not described.
		0	The objectives were partially but unclearly described
		1	Yes, the objectives were well described and clear
QA2	Does the article include a detailed description of the proposed solution or approach?	−1	No, details were missing
		0	Partially, if you wish to use the approach or solution, you must read the references
		1	Yes, the approach can be used based on the presented details
QA3	Is the proposed solution or approach valid?	−1	No
		0	It was partially validated in a laboratory, or only portions of the proposal were validated
		1	Yes, by a case study
QA4	Does the article present an opinion or viewpoint?	−1	Yes
		0	Partially because the corresponding work was explained, and the work was set into a specific context
		1	No, the paper was based on research
QA5	Has the study been cited in other scientific publications?	−1	No, no one cited the study
		0	Partially. Between one and five scientific papers cited the study
		1	Yes, more than five scientific papers cited the study.

The first item (QA1) evaluates the purpose of each study. This question was answered positively in 82% of the studies. The second point (QA2) measures whether the study presents a detailed description of the approach, and the answer to this question was positive in 77% of the studies. The third item (QA3) asks about a method of validating the result, with only 21% of studies using adequate validation methods. The fourth point (QA4) evaluates whether the studies are based on opinions or points of view. Only 31% of studies responded positively. Finally, the fifth item (QA5) looks at the number of citations received by studies, and the answers demonstrated that 53% of the studies had more than five citations in other studies.

### 3.3 Limitations

Following the quality guidelines for systematic reviews (Moher et al., 2009; Alexander, 2020), we established the inclusion and exclusion criteria, while recognizing the complexities and implications of these. We acknowledge that the review uses only three databases. This systematic review could have benefited from other databases as well as other impact indicators. We also acknowledge that the language represents another bias in the databases, as these repositories predominantly consider English-speaking articles.

Additionally, the study’s reliance on current platforms might overlook emerging technologies that could influence these domains. The exclusive emphasis on primary education raises questions about the applicability of findings across different educational levels. Cultural influences on the implementation and effectiveness of learning analytics strategies may not be fully captured. Future research should consider addressing these limitations to offer a more nuanced and widely applicable understanding of the dynamics between learning analytics and professional capita.

## 4 Findings and discussion

This systematic review aimed to address the following research questions:

### 4.1 What are the characteristics and approaches of existing learning analytics in professional capital? (RQ1)

**Figure 2:**
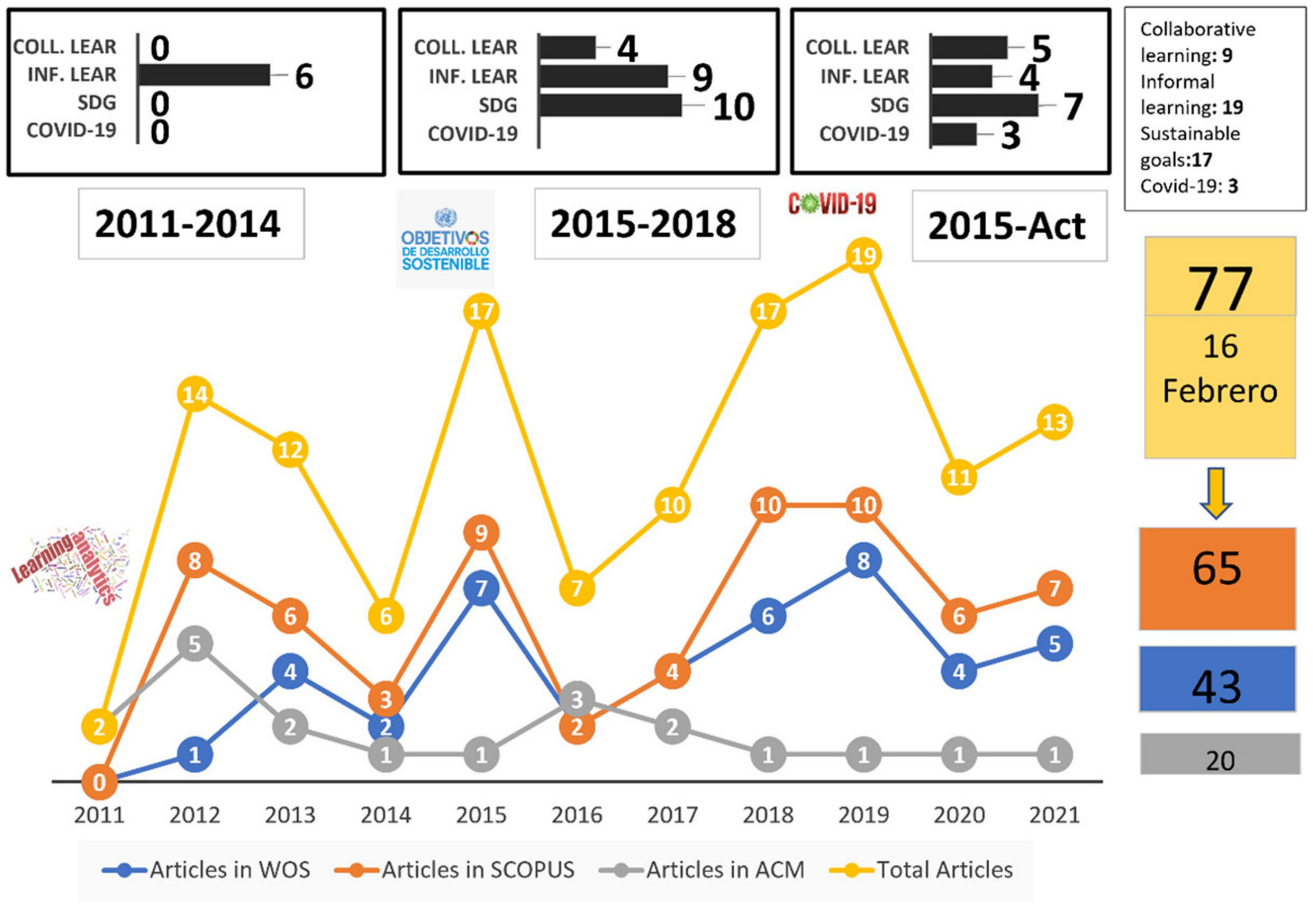
Number of accumulated publications on learning analytics in the professional capital of teachers according to source, emerging themes, and key time points.

Figure 2 reveals a significant increase in scientific production over the last ten years. In addition, these findings highlight that in the year in which the SDGs emerged (2015), and the explosion of the COVID-19 pandemic (2019), the publications peaked, with 17 articles in 2015 and 19 in 2019, although it should be noted that 2012 (the year following the appearance of LA) saw a third peak of scientific production, with 14 articles.

This Figure 2 also shows the scientific production with respect to four emerging themes in recent years. These are sustainable goals, informal learning, collaborative learning, and COVID-19, all of which have seen a considerable increase in published output from 2015 to the present, particularly Sustainable Development Goals. However, from 2011 to the present, the highest number of publications were related to informal learning (19), followed by SDGs (17), collaborative learning (9), and finally COVID-19 (3).

LA within professional capital can be seen as an indicator of improved quality and effectiveness of learning (Hu et al., 2018). However, there are other issues that can also help us understand this observation and that must be taken into account. For example, de Laat and Schreurs (2013) show that informal learning has a strong relationship with the increase in professional capital. However, as powerful as informal learning can be, a challenge arises when attempting to use it for professional development. Informal learning activities are mostly implicit, spontaneous, and invisible to others, and as such, this problem presents an interesting challenge for the LA field, that is, finding ways to capture and analyze traces of informal (social) learning in everyday life and networks (Cross et al., 2004).

Similarly, collaborative learning between professionals emerges, which can begin to generate professional capital (Hargreaves and Fullan, 2012), that is, teaching wisdom, collaboration, and mastery of the content they develop, which can extend from the cloud to the classroom. While much of learning analytics can focus on the classroom and/or educational institution, many transformations are also taking place in learning on and through the web to achieve collaborative output, and such changes are therefore equally open to inquiry from learning, network, and analytical perspectives, as a considerable amount of information can be extracted from social networks (Haythornthwaite, 2011).

Stoll et al. (2010) has demonstrated the importance of focusing on collective knowledge and growth that permeates community life. Through continuous inquiry and reflection, members are encouraged to seek new knowledge by continually examining their practices and engaging in thoughtful dialog, applying new ideas, solving problems, and finding solutions.

The concept of collaborative learning leads us to the term *network learning* (LeCun et al., 2015). This refers to the collective advancement of knowledge and the development of shared identities that come together in the community aspect of social learning, based on the well-known concept of communities of professional practice — a theme of central relevance in the collaborative learning literature in recent years (Lave, 1988). Therefore, the collaborative learning concept tends to be closely linked to the improvement of professional capital through the use of LA.

As mentioned, the results show an increase in scientific production in this area of research during the last ten years, identifying the key moments and significant events that help us understand the evolution of this production. For example, the introduction of the SDGs in 2015 coincided with a surge in published output in this field, which can be explained by the fact that the educational approach adopted to achieve the targets of Objective 4 (quality in education) involves creating collaborative capacities among teachers through the construction of professional capital.

Thus, developing these skills in preparation for a career requires a sustained and progressive growth of professional habits. The community of professional practice represents an alternative, informal way to achieve this goal (Cook et al., 2012), a term that is repeated again in the literature because it fosters a new way of learning as students observe and emulate mentors, while engaging in a cycle of “learning to be” in order to master a particular discipline (Khousa and Atif, 2018).

Subsequently, another relevant event in the trajectory of this field is the Covid−19 pandemic. This crisis has had a profound impact on education. In the context of the pandemic, professional capital has become even more important, as there have been significant changes in the way people work, along with the skills and knowledge needed to adapt to these changes. Pedroso et al. (2021) found that those school leaders who had greater professional capital were more effective in their response to the pandemic.

**Figure 3:**
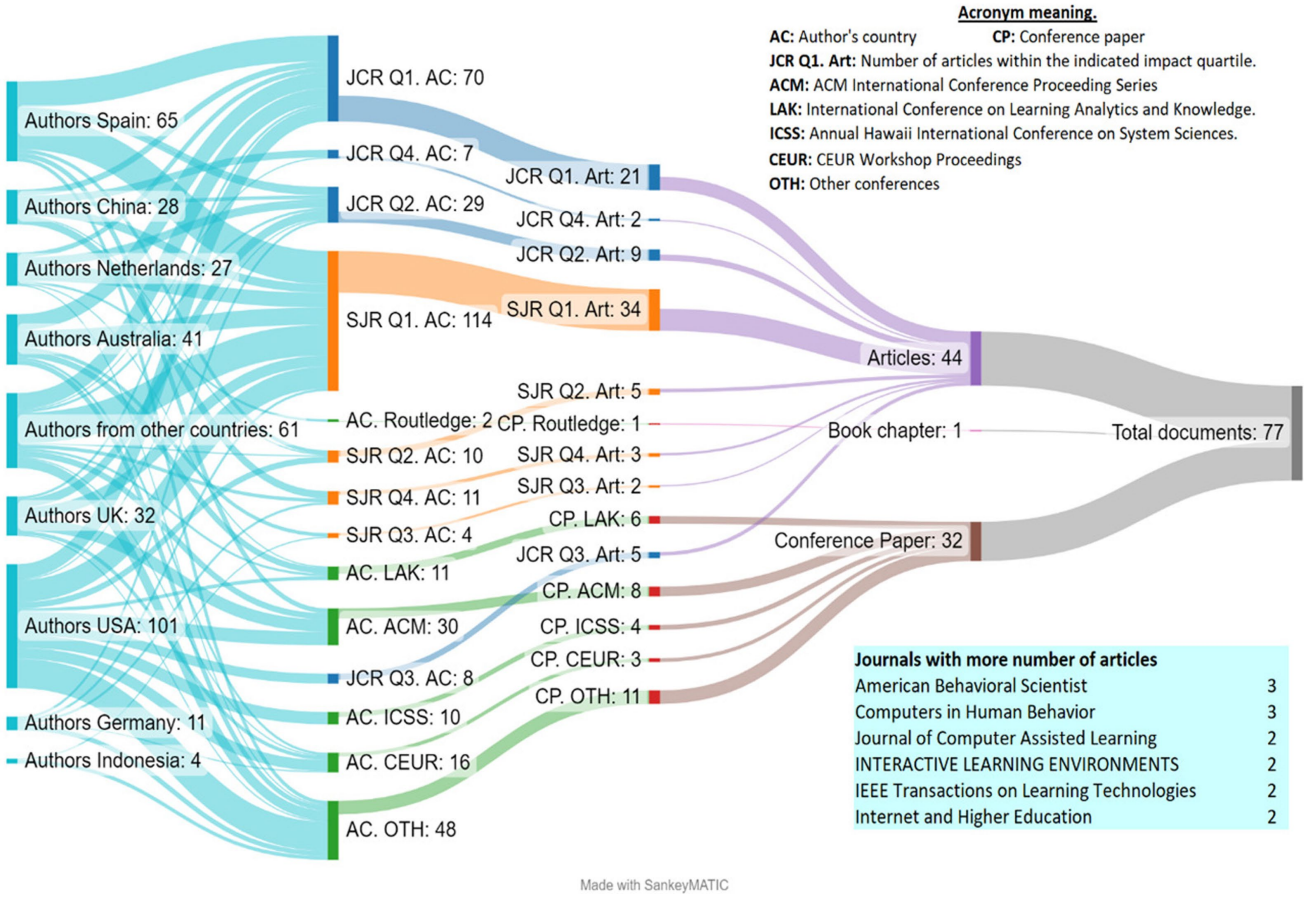
Number of publications by type of document, quartile, and author nationality.

In Figure 3 uses the Sankey diagram is used to identify how scientific production is distributed by type of document, database, quartile, and nationality of the authors.

**Figure 4:**
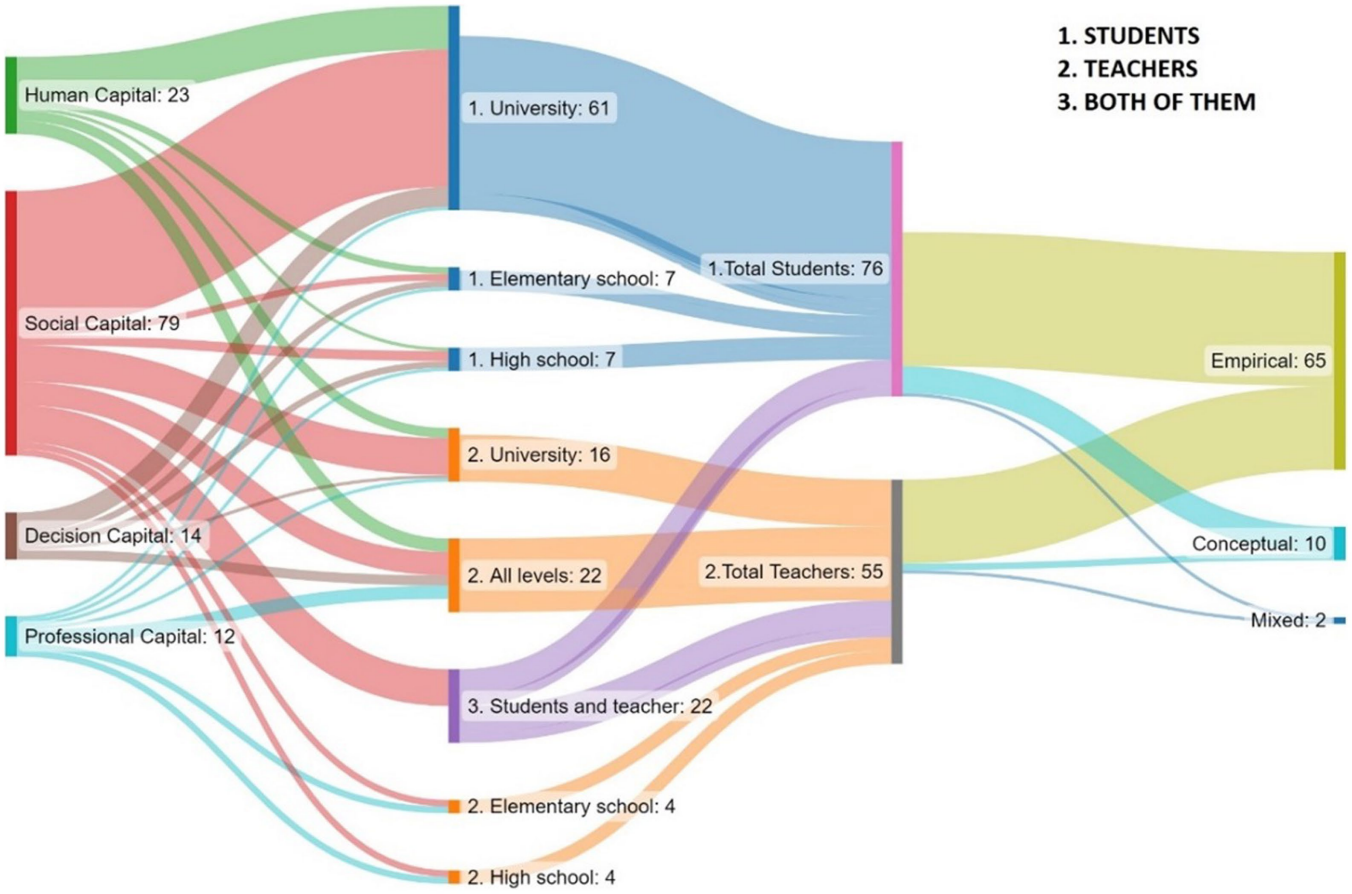
Number of publications by type of capital, level of application, and source, some studies overlap in categories.

First, moving from left to right in Figure 4, it is clear that most of the documents are journal articles (*n* = 44), followed by conference communications (*n* = 32), and only one book chapter. Of the 44 articles, most were published in Quartile 1 of the SJR (*n* = 34) and Quartile 1 of the JCR (*n* = 21), and we can see that most of the remaining articles have an impact factor. Continuing with the diagram, in terms of the number of authors per quartile based on their author’s affiliated, and in accordance with the previous findings, most of the authors appear in Quartile 1 of the JRS (*n* = 114 authors) and Quartile 1 of the JCR (*n* = 70 authors). The most predominant nationalities are the USA (*n* = 101 Authors), Spain (*n* = 65), UK (*n* = 32), China (*n* = 28), and the Netherlands (*n* = 27). Finally, the journal with the highest number of articles on the topic is American Behavioral Scientist and the *Journal of Professional Capital and Community.*

Second, regarding the 32 conference communications, the ACM Conference on Web Search and Data Mining (8 documents) and The International Learning Analytics and Knowledge Conference (6 documents) are noteworthy, where American authors account for most of the published conference proceedings, followed by the Germans and the British.

Third, only one book chapter was included (with American authorship from the Routledge publishing house).

LA is not only associated with the improvement of professional capital, but also in other areas, occupying the most relevant positions in high-impact databases. As later confirmed by Paraschiv et al. (2016), the speed at which new scientific articles are published has increased drastically, as well as the process of monitoring the most recent high-impact publications. LA is even present in the formulation of new educational policy reforms in countries such as China (Yu et al., 2016). In the United States, education and government are redefining their partnerships and working together to create competency-based, industry-driven education at the local, state, and national levels through LA methodologies (Baumann et al., 2014), which can give us an insight into the rise of specific and focused conferences on LA in the United States.

**Figure 5:**
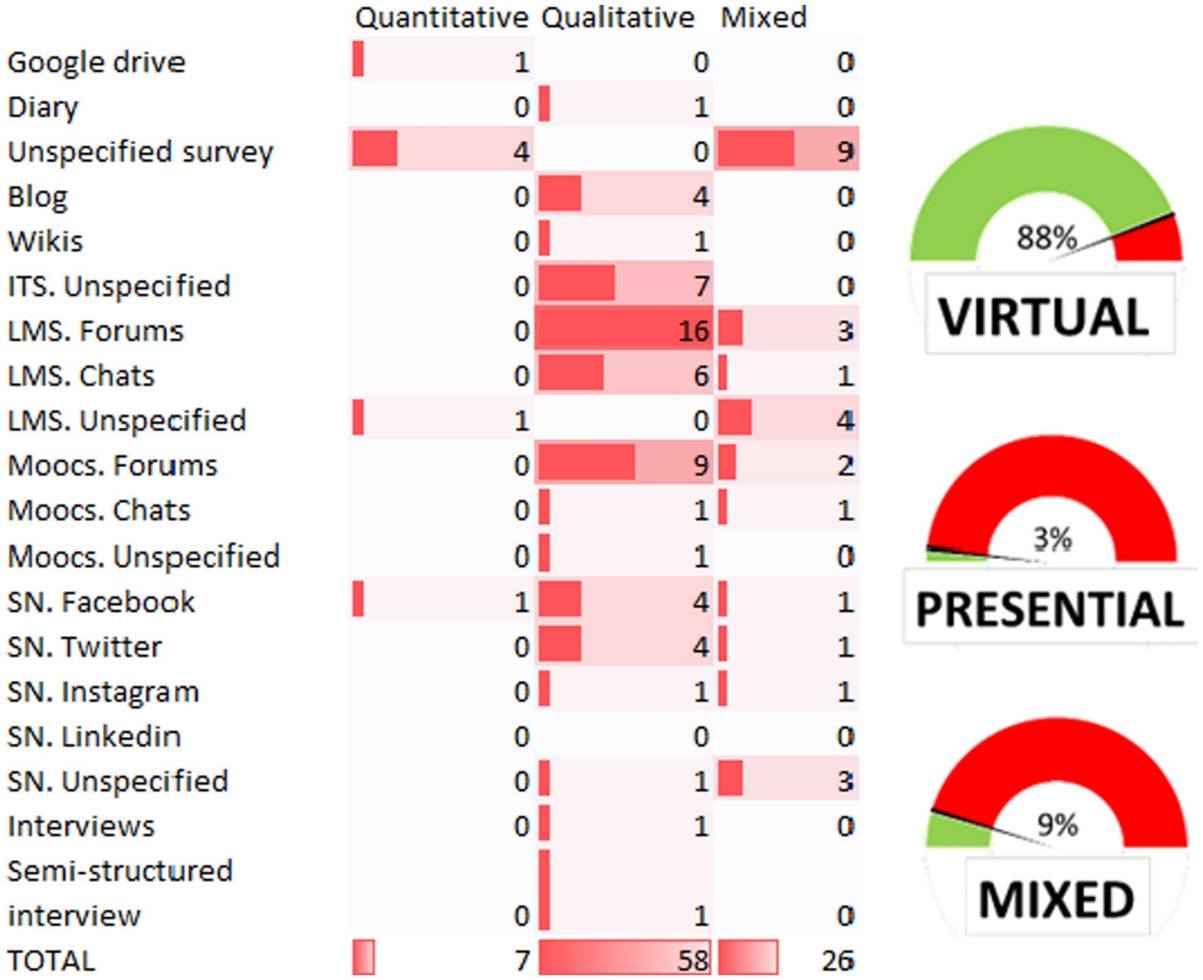
Number of publications by source of data collection and methodology used. *LMS*: ITS, Intelligent Tutoring System; LMS, Learning Management System; SN, Social Networks. The same article can have two sources of information, therefore, the total count indicated in the table does not refer to the number of articles, but to the number of times that the different sources can be used in a single article.

In line with the data shown in Figure 3, studies such as those of Monés et al. (2020), and Muñoz-Merino et al. (2022) agree in showing the increasing importance of LA in the Spanish context, in addition to its effectiveness in the use and improvement of the quality of education provided by teachers (Michos et al., 2020; Llopis-Albert and Rubio, 2021). Consequently, this is one of the focal points of Figure 5, a Sankey diagram which indicates that most of the articles are empirical (*n* = 65), followed by conceptual (*n* = 10), and mixed (*n* = 2).

Continuing in the same lines, the following categorization shows the focus of the study, i.e., whether the research focuses on students or teaching staff. On a total of 76 occasions, different groups of students are studied at varying levels, while on 55 occasions the teaching staff are the focus of the study. It should be noted that conceptual studies tend to focus on students, with a notable lack of conceptual studies focused on teachers.

The bulk of scientific output centered on the student demographic is primarily directed toward university-level students, with a shortage of research on school students. However, teaching staff studies are more balanced, addressing all levels of education. Moreover, there is a notable predominance of publications at the university level (albeit to a lesser extent than the student-focused articles), and, as noted above, there is again a lack of research related to professional capital and learning analytics at the primary education level. Finally, the articles that focus on both teachers and students address these populations together and at all levels.

The last block on the right shows the number of investigations on the various types of professional capital. At first glance, it is evident that social capital accounts for the majority of the studies, while there is a similar distribution of human, decisional, and professional (general) capital studies.

Consequently, it is evident that conceptual studies are aimed at improving social capital in students, suggesting a gap in terms of translating this work to teachers, while providing a foundation upon which productive learning can be established (Kovanovic et al., 2014).

Demir’s (2021) highlights the need for more research on the relationship between these dimensions and the organizational structure of schools to promote the desired outcomes of teacher social capital. Based on this, empirical studies show the considerable importance of social capital within the broader spectrum of professional capital (Hargreaves and Fullan, 2012), leading to the adequate exchange of knowledge and practices. These dynamics contribute to the creation of challenging learning environments that are conducive to increased creativity, thereby enabling the acquisition of fresh insights into student success.

As a result, there is a gap in terms of studies on social capital at elementary and primary levels. An argument can be made for including social capital as an explicit component of the ability of community schools to use data on student outcomes to increase student success. On this basis, several articles support Newmann et al.'s (1997) description of the organizational capacity of schools to meet performance expectations. For instance, Smylie and Evans (2006) highlight the role of Coleman’s (1988) concept of social capital for policy implementation, while studies such as those of Yoon et al. (2018) found a relationship between the presence of forms of social capital as part of organizational capacity and the frequency and extent of data usage among teachers and administrators.

In light of research on organizational learning, it appears that social capital provides opportunities for the creation of new knowledge, such as possible solutions to persistent problems of student success, and research on organizational routines as mechanisms for change and preservation in organizations (Kerrigan, 2015).

Another argument is the ability of social capital to lay the foundations for the other two types of professional capital (human and decisional). Evidence for this possibility can be found in the study by Coleman (1988), who demonstrated that the effect of social capital is especially important in the creation of human capital, while social capital in both the family and the community plays a role in creation of human capital in the next generation. In this context, universities can use learning analytics to help teachers understand and monitor pedagogical practices that are designed to build social relationships among students and actively engage them in their learning environment (Carceller et al., 2013).

LA methods can be employed to identify the potential impact of socialization efforts of active participation in “learning relationships” for professional development. In this regard, LA is aim at formulating tools to raise awareness of the presence of learning activities and processes. This enables the subsequent collection and analysis of such processes to ultimately improve activities, all of which are key elements tied to the next question to be addressed (Ferguson and Buckingham, 2012; Siemens, 2013).

### 4.2 What does learning analytics suggest should be evaluated and analyzed to improve professional capital? (RQ2)

To respond to this question, three perspectives are raised: what dynamics are most frequently used for the collection of these data? How and what are the techniques employed to extract useful information from these data? And, finally, what strategies are being employed to improve professional capital? In order to delve deeper into the first issue, Figure 5 is presented.

First, this figure visualizes the data collection methods in the form of a speedometer, showing that in 88% of the cases the data is extracted virtually, 9% use mixed methods (physical and virtual), and only 3% of the studies collect data entirely in person.

The horizontal bar in the table indicates the methodology used, while the vertical bar refers to the type of source or tool used for collection. Regarding the type of methodology, qualitative methods predominate, appearing on 59 occasions in the documents analyzed, followed by mixed methodologies (26 times), and qualitative methods (7 times).

Moving to the vertical axis, we can first observe which of the qualitative sources were used most frequently, following the categorizations presented in Figure 1. It is noteworthy that both LMS platforms (*n* = 16) and MOOCs (*n* = 9) are used for data extraction, followed by LMS chats (*n* = 6). It is also worth highlighting the homogenous distribution of data gathered from social networks (Facebook *n* = 4/Twitter *n* = 4) and blogs (*n* = 4). This is followed by mixed methodologies, where unspecified questionnaires (*n* = 9) are used together with LMSs without specifically specifying the source (*n* = 4), as in the case of social networks (*n* = 3). Finally, in the quantitative column, the data are collected from unspecified questionnaires (*n* = 4).

It is important to note the significance of the data collection method, which is useful for defining individual, school, and systematic objectives for professional development. Furthermore, this process offers invaluable insights into teachers’ learning needs, which are crucial for planning meaningful professional development initiatives (Guskey, 2002). However, a large part of the sample of articles also focuses on data gathered from students, so we must address these findings from both perspectives, even though they are directly interrelated (Harris and Sass, 2011). At the same time, it is necessary to consider that after addressing the first question, the data will focus on the social capital dimension as being key to the improvement of professional capital (Day, 2013). Consequently, these data are highly relevant as they reveal patterns that shed light on the perceived sense of community (Dawson, 2008), participation and social connections (Fournier et al., 2011), disconnected students and teachers, as well as communication between them (Macfadyen and Dawson, 2010).

Based on these findings, and after contextualizing the information, this can be used to identify the potential impact of socialization efforts via active participation in forums, which our study suggests is the main tool for data extraction, a trend supported by numerous articles concerned on improving professional capital, such as those of Poole and van de Ven (1989), Järvelä et al. (2015), and Hammond (2019). The results of these studies highlight the importance of online forums for professional development, especially regarding collaboration and knowledge sharing. Forums can encourage reflection, discussion, and shared learning, which can help teachers improve their practice and raise the quality of teaching overall. In addition, these studies show how forums can be effectively designed and evaluated to support online teacher professional development (Rocco, 2010). Therefore, this information can guide teaching and learning practices by providing an insight into the role played by online discussion forums in both online and blended modes of delivery. However, a limitation of forums, as noted in the study by Kavanaugh and Patterson (2001), is that by relying on forum data, we focus only on the comparatively small proportion of enrolled students who actively participated in them. To summarize, participation in discussion forums provides access to professional capital, which can then be used to access resources and support student outcomes, while having a positive impact on professional development (Misanchuk and Anderson, 2002; Šmite et al., 2017), leading us to encourage the creation of professional capital for quality teaching and learning.

Although there is a notable use of qualitative methods in our analyzed corpus, there are still a few papers that use mixed methods, such as the studies by Peeters and Vaidya (2016) or Saunders (2014), where they show how mixed data collection models can provide a more complete and detailed view of professional capital. By combining quantitative and qualitative methods, objective and subjective data can be collected to provide a deeper understanding of teaching practice and the impact of professional training and development (Carson et al., 2020; Gidari and Kakana, 2022). In addition, these mixed models can help address the limitations of individual data collection methods and improve the validity and reliability of results (Fetters, 2016).

**Figure 6:**
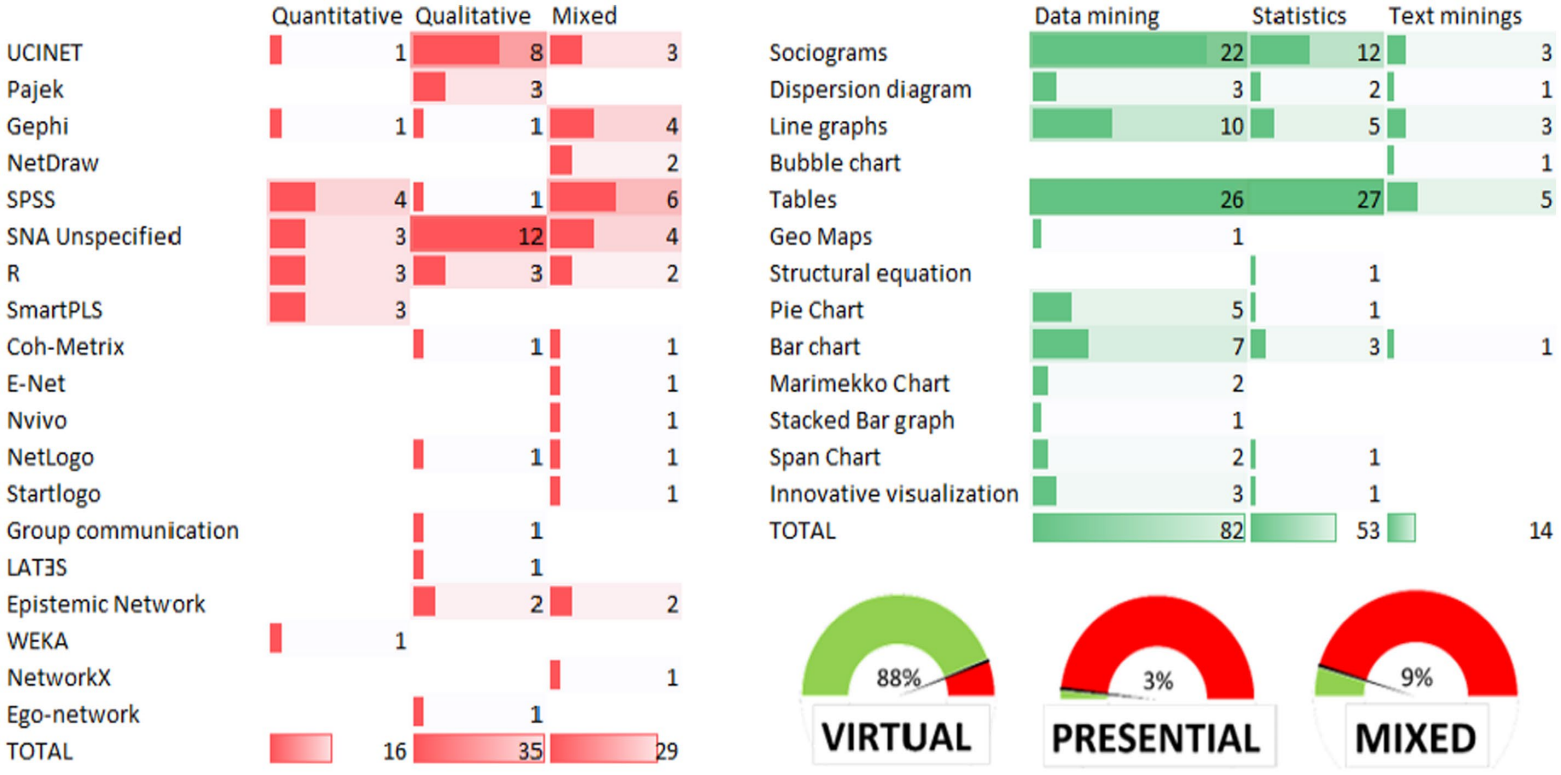
Number of publications according to data source, methodology, analytical method, and visualizations used. The same article can have two visualizations or information analysis tools. Therefore, the total count indicated in the table does not refer to the number of articles, but to the number of times that the various tools or sources can be used in an article.

Figure 6 also shows a scarcity of primary usage of quantitative methods. This finding aligns with the research described by Zhang (2022) who pointed out that the data on professional capital should be collected through mixed methods that combine quantitative and qualitative techniques. Quantitative data collection can provide useful information, but it cannot capture the full complexity of teaching practice, as teaching and learning processes are multifaceted and contextual (Ross and Bruce, 2007). Therefore, it is also necessary to use qualitative methods to understand the experience and perspective of teachers in the classroom and in their professional environment.

In addition, the use of a single quantitative method can lead to a fragmented and incomplete view of professional capital, as it does not take into account the context in which learning and teaching occurs. Thus, there is the need for a more holistic approach that integrates various sources of information and perspectives. In general, these studies indicate that to understand professional capital properly, it is necessary to use mixed research techniques that combine quantitative and qualitative methods (Popham, 1999).

However, it is imperative to explore other methods of data collection, such as the direct observation of individuals, as employed in the study by Hu et al. (2018), which captured most of the typical learning experiences of students in the classroom. In addition, techniques that facilitate the management of data from collection to analysis are increasingly being used. These systems include MySQL (Bieke and Maarten, 2012), an open source relational database management system, which in practice can help improve efficiency in the management of information of teaching staff, which allows more time for teaching and less time for administration or monitoring student attendance and academic performance (Paredes and Chung, 2012).

Having identified the relevant aspects of LA in the collection of evidence on professional capital, the next analytical step is to attempt to transform the findings into ordered and meaningful knowledge that allows conclusions to be drawn, for which the results shown in Figure 6 were used.

Generally speaking, social learning analysis provides a methodological lens for making sense of these massive data streams (Ahn, 2013). Detailed inspection of the results confirms that within professional capital research, the most used category of analysis within learning analytics is network analysis. This allows detecting patterns of behavior and learning communities (Hernández and Navarro, 2018), while identifying the most influential students and teachers within a group to design pedagogical strategies tailored to the specific needs of each of them.

First, a speedometer at the bottom-right part of the figure shows the origin of the type of data to be analyzed (virtual, face-to-face, or mixed). This figure shows that in 88% of the cases, data were extracted from a virtual sample, 9% were mixed (a mixture of physical and virtual methods), and only 3% of the studies collected data entirely in person.

Next, two tables are presented. The table with red bars compares the analytical tools used (vertical bar) in the studies with the type of methodology used (horizontal bar), while the table with green bars shows the visualization used (vertical bar) to show the data after the various analyses were performed (horizontal bar).

The red table indicates that the most frequently used methodology is qualitative (*n* = 35), followed by mixed (*n* = 29), and quantitative (*n* = 16). Following this rationale, for purely qualitative methodologies, the most widely used instrument is UCINET (*n* = 8), followed by R and Pajek (*n* = 3 for both), while in 12 occasions the analysis tool was unspecified, which is a limitation of our study. For hybrid methodologies, there is a notable use of SPSS (*n* = 6), followed by Gephi (*n* = 4), and Ucinet (*n* = 3). Finally, for the quantitative methodologies, homogeneous results appear, with the use of tools such as SPSS (*n* = 4), R (x3), and SmartPLS (*n* = 3).

This table also shows that l data mining is the most frequently used analytical technique (*n* = 82), followed by statistical techniques (*n* = 53) and, in a limited number of cases, text mining (*n* = 14). In the same order, the use of tables as a visualization method is predominant within data mining (*n* = 26), followed by sociograms (*n* = 22), line graphs (*n* = 10), bar graphs, and pie charts. Of the studies using statistical procedures, tables are again the most frequently used (*n* = 27), followed by sociograms (*n* = 12) and line graphs (*n* = 5). Finally, a similar distribution was found for text mining studies, with the predominant use of tables (*n* = 5), followed by sociograms (*n* = 3), and line graphs (*n* = 3).

Therefore, as this study demonstrates, network analysis is the most used technique to analyze data related to professional capital because it allows us to understand the complexity of the relationships and connections between teachers and colleagues, students, parents, as well as other stakeholders within the educational system. This approach is evident in the research of Bryant et al. (2017), where social capital is used to facilitate the resolution of complex problems in a large and interdisciplinary team, or Chapman et al. (2016), who conducted a collaborative research and found that schools reported greater evidence of an impact on positive outcomes for disadvantaged students. In addition, the need to use these types of techniques has increased in the wake of the COVID-19 pandemic, as seen in studies such as those of Raaper and Brown (2020).

A key tool within this branch of LA is UCINET (Borgatti et al., 2002), a social network analysis software that is widely used in social and organizational research. This instrument is used to analyze and visualize social networks and provides tools to study the relationships between actors, the flow of information, and the characteristics of the network itself. Therefore, UCINET appears to be a reliable social media tool in the educational field for the analysis of professional capital (Wang et al., 2017). This approach could be particularly useful for those seeking to understand professional capital and how it relates to success at work. For example, this instrument helps educators to reflect on whether their individual social networks are sufficient to allow cooperative actors to be willing to share useful knowledge and/or whether cooperative culture can maintain and further strengthen enhanced creativity (Sam Liu, 2017). It is also possible to identify opportunities to expand the network and connect with influencers (Tahmasebi and Askaribezayeh, 2021).

However, in recent years, scientific articles have provided evidence to support the validity of using hybrid methodologies to conduct analyses in professional capital (Leong and Ibrahim, 2015). Nonetheless, UCINET reappears in the column of hybrid procedures, which in turn suggests that this instrument is one of the most reliable in this field. Indeed, Alwafi (2021) emphasizes the importance of using mixed methods to study professional capital on Twitter.

Nevertheless, SPSS and R have also become more present in mixed methods (Muenchen, 2011), although they feature most centrally in quantitative analysis and are accompanied by other programs when the qualitative component comes into play. However, in recent years, R has become more present in qualitative works, as shown in studies such as Oliveira e Sá and de Castro (2020), where R is used qualitatively to analyze leadership, collaboration, and reflexivity from a dialogic and innovative perspective with the aim of improving professional development. Jan et al. (2019) states that while network analysis is effective in detecting key participants, subgroups, and certain aspects of a community of practice, a specific measure of network analysis cannot be correlated with a particular presence in a research community. Therefore, network analysis should be complemented by a qualitative analytical technique.

It is also unsurprising that the combination of *data mining* and statistics frequently appears in our sample of studies, since they allow for analyzing large datasets and extracting significant patterns and relationships, which can be very useful in decision-making and the development of strategies for promoting professional capital. For example, Xing and Gao (2018) discuss the relationship between online discourse and engagement in Twitter’s professional learning communities, and by analyzing the most relevant topics in conversations, these authors gained an insight in the perception that users have about a person and opportunities for collaborations, purely through the use of data mining techniques and statistics.

**Figure 7:**
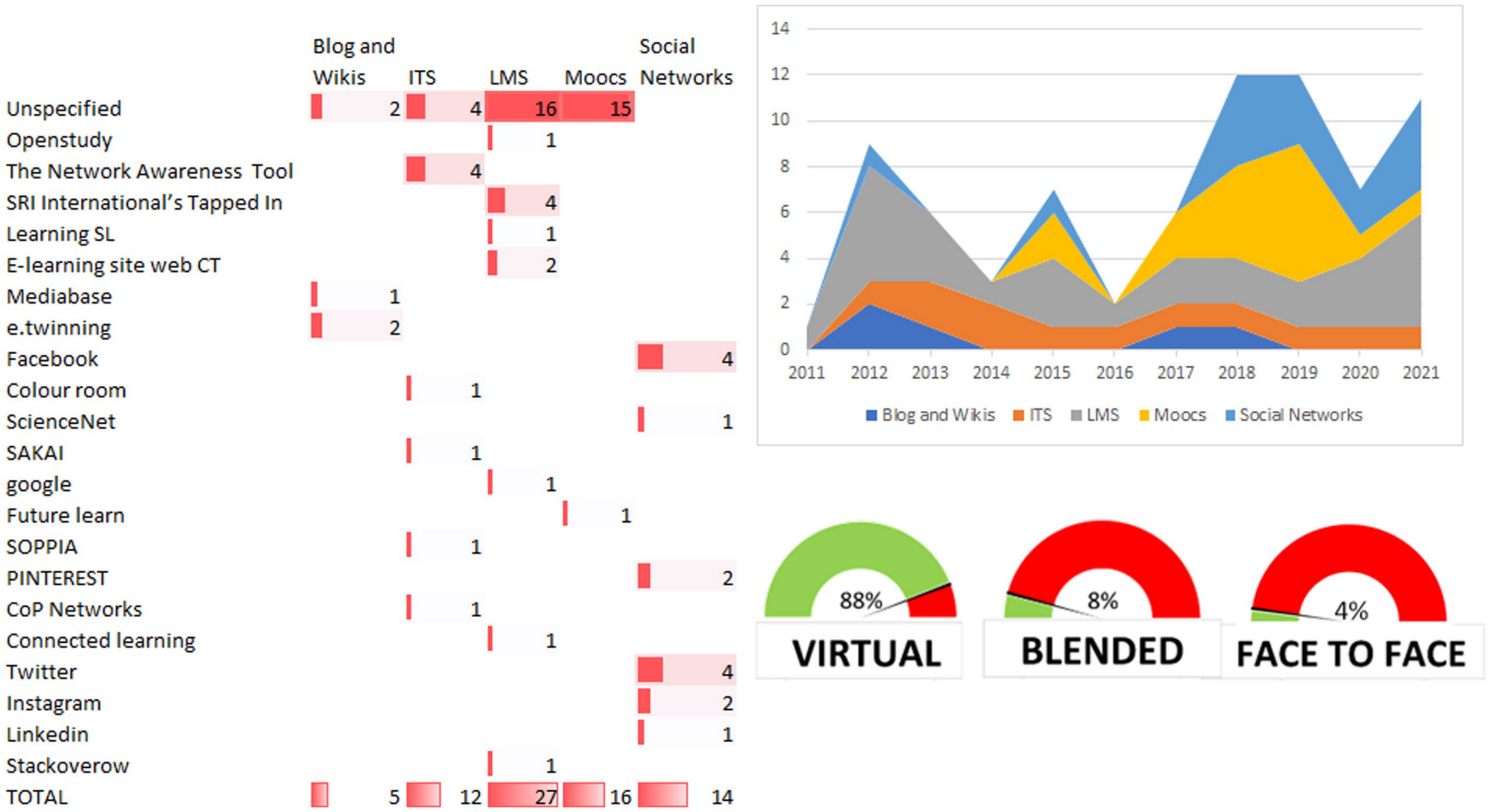
Programs, platforms, and type of intervention for the improvement of professional capital, as well as its evolution over time. *LMS*: ITS, Intelligent Tutoring System; LMS, Learning Management System; SN, Social Networks. The same article can have two improvement platforms, and therefore the total count indicated in the table does not refer to the number of articles but to the number of times that the different platforms can be used in an article.

The visualizations of these data assume a relevant role within LA (Vieira et al., 2018), and in this regard, sociograms emerge as one of the most used strategies for facilitating the presentation of data to interested parties (Tubaro et al., 2016) and have a long tradition in the field (Keithlucas, 1957). Because sociograms provide a clear representation of the relationships and connections between teachers in a network while establishing the roles and positions of teachers therein (Ferreira et al., 2018), they help identify opportunities for collaboration and professional development among teachers (Tatum et al., 2013). Moreover, they facilitate the identification of gaps in professional capital and possible solutions (Loomis, 1948), along with the monitoring and evaluation of professional capital over time (Atkinson, 1949). Network analysis also facilitates the identification of the most effective strategies for improving the quality of teaching and the evaluation of the impact of professional development policies and programs on students and teachers (Zhao, 2009). This concept is central to the content presented in Figure 7.

To conclude addressing this research question, Figure 7 shows the programs and types of platforms used for improving professional capital, as well as the evolution of the latter variable over time.

On the bottom right of the figure, a speedometer shows the intervention modality (virtual, blended, or face-to-face). In 88% of cases the intervention is implemented virtually, 8% used a blended intervention, while only 4% used a face-to-face modality.

Moving to the table with the red bars, the columns show the type of platform used for the intervention, while the s rows indicate the specific name of the service used for the intervention. Regarding the type of platform, the use of LMS and MOOCS (*n* = 27 and *n* = 16, respectively) are notable, followed by social networks (*n* = 14), ITS (*n* = 12) and finally blogs and wikis (*n* = 5). The most prominent LMS of the rest is SRI International’s Tapped In (*n* = 4). MOOCs have unspecified names, while the most used social networks are Facebook and Twitter (both n = 4). Within ITS, we can highlight The Network Awareness Tool (*n* = 4) and finally e.twinning (x2) within blogs and wikis.

On the temporal exploration of the included articles, from 2011 to 2013, there was a predominant use of LMS (without the presence of MOOCs and social networks), peaking in 2012 with a total of twelve appearances. The period of 2014–2017 saw the lowest volume of scientific production, peaking in 2015 with a total of 7 cases in which these services were used for improving professional capital. It should also be noted that the use of MOOCs appears, but to a much lesser extent. Finally, 2017 to 2021 represents the most significant period, where the field appears to be emerging, reaching a peak number of scientific publications to indicate the important role played by MOOCs and the use of social networks.

First, and according to the results, LMSs emerge as the most prevalent and consistently employed platforms. This preference is due to their robust features, including organized learning management, planning, evaluation tools, communication, and collaborative capabilities. These attributes make LMSs an ideal form of intervention in the quest to promote professional capital. These ideas are supported by studies such as that of de Laat and Schreurs (2013), where LMSs are used to implement an approach to professional development that is connected to the informal day-to-day networking activities in the workplace, while providing instructions to develop automated and scalable LA tools that facilitate the establishment of informal networks to better leverage their learning potential.

However, it should also be noted that LMSs have a considerable impact when used with social network integration. Some studies show that although students like to use Facebook as an LMS, many of them find as many advantages as disadvantages when compared to a traditional LMS such as Moodle (Al-Dhanhani et al., 2015). Facebook is clearly preferred by students to communicate instantly with their teachers and participate in discussions, but not for sharing materials and submitting assignments (Verdu et al., 2021).

Regarding the intervention platforms used to improve professional capital, the emergence of MOOCs has become evident, and in recent years these have formed a central axis for collaborative work between students and teachers, as shown by the considerable increase in scientific production in this specific area. This notion is supported by the literature review of Zhu et al. (2022), who analyzed 166 articles between 2011 and 2021 to summarize the trends and critical problems of integrating LA in MOOCs, revealing that this approach was more often used for research purposes than in practice (i.e., learning and teaching). In addition, approximately 60% of the articles adopted student registration data, which also indicates a trend toward the gap found in our study, where most of the articles have focused on students rather than teachers.

Consequently, some of the benefits brought to the field include the ease of keeping up with the latest advances in their field and improving their teaching practice, and the greater flexibility offered in terms of schedules and pace of learning, allowing educators to take online courses and training in their own time and place. Moreover, educators are given the opportunity to become familiar with the latest online teaching technologies and tools (Rincón-Flores et al., 2019) and have the chance to interact and collaborate with other educators around the world, which can provide new perspectives and enrich teaching practices (Paton et al., 2018).

In this regard, studies such as that of Solórzano-García and Navío-Marco (2019) show how Moocs generate knowledge through the increase of professional capital, which has an impact on the improvement of learning communities through the recognition of other members. In addition, it is worth highlighting the algorithms inserted within MOOCs, such as the Latent Dirichlet Allocation (LDA), which is used to recognize latent knowledge (Zarra et al., 2018). Therefore, it is clear that the integration of social networks, algorithms, or blogs within the same platform seems to be one of the most effective interventions, while the isolated application of a single service on the same platform generates difficulties and gaps in learning (Koedinger et al., 2015).

As for social media, the surge in its use over the same time period appears to be undeniable, and this emerging field reveals information about how teachers gain experience, the effectiveness of leadership structures, how councils, professional, and personal networks support teaching, and why and how school reforms spread across districts (Leana, 2011; de Laat and Schreurs, 2013; Spillane et al., 2015; Yoon et al., 2018). This critical mass of research on the potential of social capital and social networks to impact educational contexts has encouraged scholars and school leaders to move toward research and intervention design informed by the findings.

However, there are few studies of these interventions, particularly with regard to how they have been implemented, what mechanisms promote change, and, most importantly, how the interventions have improved teaching and learning. We need to have clear models and strategies for implementing those interventions, and in the following studies, we show some specific examples of the use of social networks today.

The study by Subekti et al. (2019) assessed the compatibility of collaboration between teachers through interactions on their social networks using the Weight Decision Matrix (WDM) algorithm. Lu et al. (2020) used social networks to identify hidden patterns in the network, finding isolated networks and attempted to unite them; while Oktavia and Sujarwo (2020) used information gathered from the social media platform to generate recommendation systems from learning partners that can provide suggestions for educational institutions.

Follow-up studies of the groups examined should also be conducted to investigate how social capital dynamics evolve over time and how they contribute to the creation of group identity (Ranieri et al., 2012).

Given the results of this research, it is logical to focus on the improvement of social capital as a key to the creation of professional capital, since it provides opportunities for the generation of new knowledge, such as possible solutions to persistent problems of student and faculty success, and research on organizational routines as drivers of change and preservation in organizations. To achieve these goals, it is necessary to identify the most influential variables in social capital as a mechanism to design the most effective interventions adapted to each context and needs, as well as the learning analytics studies involved, all of which will be the focus of our next and final research question.

### 4.3 What is the most untangled dimension of learning analytics in professional capital, and how learning analytics is improving this dimension? (Q3)

Our findings from the first research questions shows that social capital is the largest area untangled by the field of learning analytics (see Figure 4). In this section, we will further explore the variables through which learning analytics is enhancing social capital within the professional capital context.

**Figure 8:**
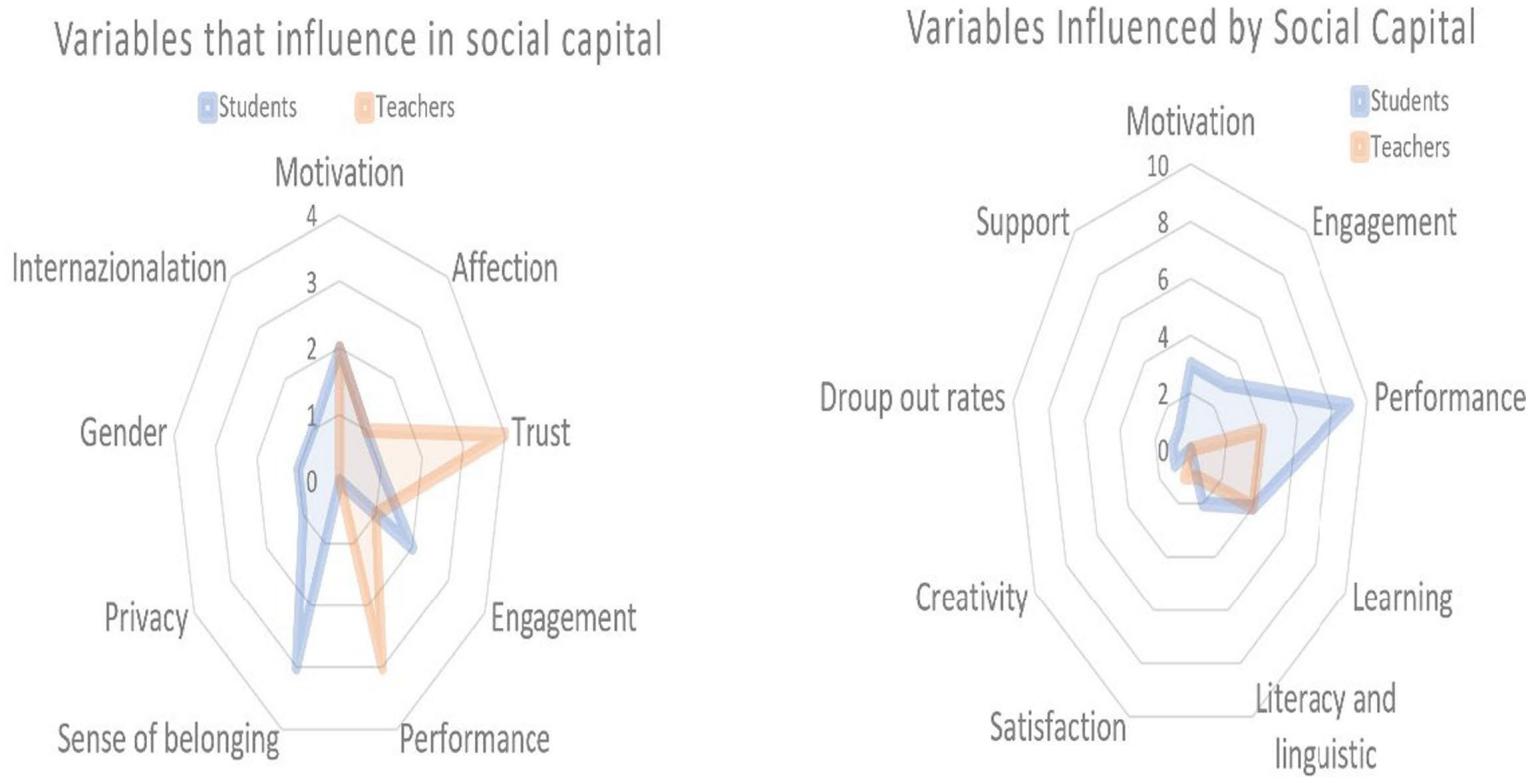
Number of publications according to variables that influence and are influenced by social capital and grouped according to whether the studies are based on students or teachers. The count refers to the number of articles that address the dimensions mentioned.

To answer this, it is necessary to analyze the articles that address social capital in a specific way, to observe which variables are of influence and are influenced. To facilitate this task, a spider diagram was created (see Figure 8 and accompanying notes to understand the numbers) to help clarify the most significant variables involved in social capital. The right-hand panel shows the variables that influence social capital, while the left panel shows the variables influenced by social capital, both divided according to whether these refer to students or teachers.

Beginning with the variables that influence social capital with respect to the student, the feeling of belonging to the community appears as the most influential (with a total of 3 articles), followed by commitment and motivation (*n* = 2 each). Concerning teachers, the trust dimension is the most predominant object of study with a total of 4 articles, followed by performance (*n* = 3), motivation (*n* = 2), and finally commitment, with a single article.

On the other hand, the variables influenced by social capital according to studies conducted in students include various aspects of performance with a total of 9 articles, followed by learning (*n* = 4), and motivation and commitment (*n* = 3 for each). Regarding the teaching staff, there is a lack of scientific production compared to that related to students, since only 4 works are concerned with learning and performance as dimensions influenced by social capital.

In this regard, this study supports works such as that of Moolenaar et al. (2012), in which social capital appears as a variable that can increase professional capital. However, we need to take into account the variables that support social capital in this process, such as the sense of belonging among students, because people who do not feel part of a group may feel isolated, disconnected, and less committed, while those who feel that they belong to a group are more likely to feel engaged and connected to other members of the group. In our work, this commitment is also shown to be an influential variable in social capital.

As explained, Ahn and Davis (2020) provide solid evidence to show how the sense of belonging and social capital are theoretically and empirically intertwined, and similarly, Cheung (2011) states that people who feel part of a group may also be more willing to collaborate in community projects, contribute ideas and resources, as well as help the other members of the group. Finally, results such as those reported by Glass and Gesing (2018) agree with our study regarding the importance of a sense of belonging and commitment as decisional variables in social capital.

This work on social capital in students has reported improvements in student performance, as supported by the data of our review, along with studies such as those of Salimi et al. (2022) demonstrating that social and personal integrative benefits play a mediating role in the relationship between online social capital and academic performance.

From the perspective of teachers, trust between colleagues emerges as a key aspect requiring attention, since this lays the foundations for increasing social capital. Indeed, when trust is high, people take a step forward voluntarily and work together in an efficient and optimal manner. As Allan and Persson (2020) state, they embrace a common purpose, take risks, think creatively, help each other, and communicate openly and sincerely.

Based on the works just described, there appears to be a difference between students and teachers in terms of the volume of work conducted on social capital, with a greater amount of work dedicated to students. Therefore, the need arises for the teachers themselves to collaboratively work in the classroom and as agents involved in the community.

It appears to us that trust, motivation, and social connections are intertwined and, consequently, require greater consideration. For example, Nahapiet and Ghoshal (1998) analyzed the social concept of ‘intellectual capital ‘by which they refer to the knowledge of a social community, such as an organization or professional practice groups. Thus, a key question is how can we create the motivation and confidence to sustain a spiral of construction of intellectual and social capital in networked practices?

This response must be based on the formation of Communities of Professional Practice with a shared purpose. In fact, numerous studies in our sample directly relate social capital to communities (Solórzano-García and Navío-Marco, 2019; Alwafi, 2021). It can be noted, then, that the concept of social capital has provided researchers and education practitioners with valuable insights into community building (Bryk and Schneider, 2002; Penuel et al., 2013). Communities of professional practice create social capital by providing opportunities for professional interaction and dialog to improve teaching practices. A leader can develop the collective capacity of a staff to achieve better results by ensuring that teams use collaborative time to engage in dialog and processes that positively impact student learning.

In short, the variables analyzed by LA that support social capital in this study will give us guidance on how to work effectively to improve professional capital, all of which involves creating communities that promote a shared identity within a network of people as well as the collective development of a particular domain or theme.

Methodologically, our study offers valuable insights by scrutinizing the diverse data collection strategies employed in learning analytics for professional capital improvement. The emphasis on forums and tools provides not only a comprehensive overview but also serves as a practical guide for educators seeking effective ways to harness the potential of data.

Interdisciplinary collaboration takes center stage in our discussion, highlighting its pivotal role in elevating the rigor of studies. We advocate for mixed methods analysis, emphasizing the importance of collaborative efforts to enrich the exploration of learning analytics and professional capital.

Furthermore, we recommend to explore MOOCs and learning analytics interventions, to incorporate automated community discovery techniques, and to adapt to the challenges posed by the COVID-19 pandemic, as points to serve as guideposts for scholars seeking to advance the field (learning analytics in professional capital) and address emerging issues. In essence, this study not only sheds light on the current state of learning analytics and professional capital but also lays the groundwork for a future research agenda that is both informed and innovative.

## 5 Conclusion

This study provides a systematic review of the intersection of learning analytics and professional capital. Three databases were searched and prompted the following conclusions:

### 5.1 On characteristics and approaches of learning analytics within professional capital

LA for Professional Capital Improvement is an effective line of work. LA helps to understand how to identify and establish indicators and lines of action, which can lead to collaboration with schools, their management teams, and the local community, as well as with education authorities, networks and professional associations. Our analysis revealed that collaborative learning not only takes place in formal contexts, but also occurs informally (Shepard et al., 2018), and even takes place in networks.

Increasing professional capital requires understanding how educational institutions are shaped, articulated, and productively optimized as a community for educational success. LA provides an overview of the stock of social and professional capital used by communities. In the context of the pandemic, professional capital has taken on even more important because of significant changes in the way people work and the skills and knowledge needed to adapt to these changes.

Social capital serves as the foundation for the other two types of professional capital (human and decisional capital). This form of capital plays a pivotal role in creating opportunities for the acquisition of new knowledge. While it is clear that conceptual studies have predominantly focused on improving the social capital of students, there is an evident gap in our understanding of social capital among teachers, particularly within primary education. Addressing this gap is essential for cultivating productive learning environments (Kovanovic et al., 2014).

The field analyzed occupies very predominant positions in high impact databases. This indicates a tendency to plan future research in different areas and themes. Most of the authors working in this line of research are Spanish.

### 5.2 Learning analytics uses the following data collection, analysis, and improvement strategies to increase professional capital

The forum is the main tool for extracting data on professional capital. The use of the UCINET tool appears to be significant and allows the identification of the training and professional development needs of teachers (Zhao, 2008), as well as the consequent design of strategies to improve the quality of education through data mining techniques, all with the help of visualizations such as sociograms.

The LMS appears to be the most predominant and consistently used platform over time, although it should be borne in mind that MOOCs and social networks have become a central axis for collaborative work between students and teachers in recent years. These environments offer flexibility and a variety of themes, fostering interactivity while boasting a large data storage capacity. The key methodology for this particular field seems to involve knowledge of techniques, tools, and platforms for social network analysis. However, few studies have focused on informing the design and implementation of intentional frameworks for creating social capital of teachers through the development of social networks (Yoon et al., 2018).

In general, the outcome of interventions through the use of platforms and web services is a useful means of making recommendations to teachers. These make it possible to suggest projects and contacts, and help build communities as well as select their members, thus maximizing social capital (Pham et al., 2012)

### 5.3 Social capital stands out as the most studied dimension within the intersection of learning analytics and professional capital

Students who feel they belong to a group are more likely to feel engaged and connected to other members of the group. This translates into improved performance and confidence among co-workers. This is therefore a key aspect to develop when aiming to increase social capital.

In general, this research has provided new insights into the current state of how a specific field — such as Learning Analytics — can help to understand how the processes of building professional capital in education are collected, analyzed, and improved. All these findings point to a number of guidelines and implications for the quality of professional capital training:

There is a need for teachers themselves (and other relevant stakeholders) to undertake collaborative classroom work. These efforts must be based on the formation of communities of practice united by a shared purpose, aligned with the goals set out in the 2030 agenda. The utilization of LA helps to restore equity in learning, which suffered significant setbacks during the pandemic period.

The analysis for the improvement of professional capital should be carried out using mixed methods. These analyses should stimulate awareness, develop networking skills, and provide information on learning outcomes in learning networks. Professional development within an organization — specifically primary education — is severely affected by a lack of scientific output. This gap suggests another promising avenue of research, that is, to examine the current themes that are giving rise to professional collaborations.

Finally, it is suggested that future studies be undertaken on MOOC and LA interventions to improve learning and teaching practices. Active interdisciplinary collaboration increases the rigor of studies and the dissemination of knowledge. In addition, we stress the need to include automated community discovery techniques in e-learning environments to facilitate and enhance their use. We also emphasize the urgency of conducting further advanced research to uncover other hidden opportunities (Yassine et al., 2022).

In short, identifying the strategies involving learning analytics within the context of professional capital is presented as a powerful approach toward developing an understanding of knowledge about networks. This, in turn, has a positive impact on the overall quality of education. Consequently, this theme represents a novel focal point for delving into the underlying reasons and objectives behind the improvement of social capital.

## Author contributions

JH-R: Writing – original draft, Writing – review & editing. MK: Writing – original draft, Writing – review & editing. JD: Writing – original draft, Writing – review & editing. QL: Writing – original draft, Writing – review & editing.

## Footnotes

^1^ https://webofscience.com

## References

[ref1] Ahn J. (2013). What can we Learn from Facebook activity? Using social learning analytics to observe new media literacy skills. Proceedings of the Third International Conference on Learning Analytics and Knowledge, Leuven, Belgium.

[ref2] Ahn M. Y. Davis H. H. (2020). Sense of belonging as an indicator of social capital. Int. J. Sociol. Soc. Policy 40, 627–642. doi: 10.1108/IJSSP-12-2019-0258

[ref3] Al-Dhanhani A. Mizouni R. Otrok H. Al-Rubaie A. (2015). Analysis of collaborative learning in social network sites used in education. Soc. Netw. Anal. Min. 5:303. doi: 10.1007/s13278-015-0303-z

[ref4] Alexander P. A. (2020). Methodological guidance paper: the art and science of quality systematic reviews. Rev. Educ. Res. 90, 6–23. doi: 10.3102/0034654319854352

[ref5] Allan J. Persson E. (2020). Social capital and trust for inclusion in school and society. Educ. Citizensh. Soc. Justice 15, 151–161. doi: 10.1177/1746197918801001

[ref6] Alwafi E. (2021). Tracing changes in teachers’ professional learning network on twitter: comparison of teachers’ social network structure and content of interaction before and during the COVID-19 pandemic. J. Comput. Assist. Learn. 37, 1653–1665. doi: 10.1111/jcal.12607, PMID: 34903905 PMC8657354

[ref7] Atkinson G. (1949). The Sociogram as an instrument in social-studies teaching and evaluation. Elem. Sch. J. 50, 74–85. doi: 10.1086/459108

[ref8] Baumann T. Harfst S. Swanger A. Saganski G. Alwerfalli D. Cell A. (2014). Developing competency-based, industry-driven manufacturing education in the USA: bringing together industry, government and education sectors. Procedia Soc. Behav. Sci. 119, 30–39. doi: 10.1016/j.sbspro.2014.03.006

[ref9] Becker G. (2010). Human capital: a theoretical and empirical analysis, with special reference to education.

[ref10] Bieke S. Maarten D. L. (2012). Network awareness tool—learning analytics in the workplace: detecting and analyzing informal workplace learning. Proceedings of the 2nd international conference on learning analytics and knowledge, New York, NY. 59–64.

[ref11] Bolívar A. Domingo J. (2023). Comunidades de práctica profesional y mejora de los aprendizajes. Análisis y estudios 74

[ref12] Borgatti S. P. Everett M. G. Freeman L. C. (2002). Ucinet for windows: Software for social network analysis. Harvard, MA: Analytic Technologies.

[ref13] Bryant L. H. Freeman S. B. Daly A. Liou Y.-H. Branon S. (2017). Making sense: unleashing social capital in interdisciplinary teams. J. Profes. Capital Commun. 2, 118–133. doi: 10.1108/JPCC-01-2017-0001

[ref14] Bryk A. S. Schneider B. (2002). Trust in Schools: a core resource for improvement. Manhattan, NY: Russell Sage Foundation.

[ref15] Carceller C. Dawson S. Lockyer L. (2013). Improving academic outcomes: does participating in online discussion forums payoff? Int. J. Technol. Enhanced Learn. 5, 117–132. doi: 10.1504/IJTEL.2013.059087

[ref16] Carson R. L. Kuhn A. P. Moore J. B. Castelli D. M. Beighle A. Hodgin K. L.. (2020). Implementation evaluation of a professional development program for comprehensive school physical activity leaders. Prev. Med. Rep. 19:101109. doi: 10.1016/j.pmedr.2020.101109, PMID: 32489771 PMC7260586

[ref17] Chapman C. Chestnutt H. Friel N. Hall S. Lowden K. (2016). Professional capital and collaborative inquiry networks for educational equity and improvement? J. Prof. Cap. Community 1, 178–197. doi: 10.1108/JPCC-03-2016-0007

[ref18] Cheung C. (2011). Children’s sense of belonging and parental social capital derived from school. J. Genet. Psychol. 172, 199–208. doi: 10.1080/00221325.2010.520362, PMID: 21675547

[ref19] Coleman J. S. (1988). Social Capital in the Creation of human capital. Am. J. Sociol. 94, S95–S120. doi: 10.1086/228943

[ref20] Cook J. Schmidt A. Kunzmann C. Braun S. (2012). The challenge of integrating motivational and affective aspects into the design of networks of practice. CEUR Workshop Proceedings, 957. https://www.scopus.com/inward/record.uri?eid=2-s2.0-84924874062&partnerID=40&md5=1c0f3aa53642d91aa8b58bc4c1cbe62e

[ref21] Cross R. L. Parker A. Cross R. (2004). The hidden power of social networks: Understanding how work really gets done in organizations.

[ref22] Daly A. J. Moolenaar N. M. Bolivar J. M. Burke P. (2010). Relationships in reform: the role of teachers’ social networks. J. Educ. Adm. 48, 359–391. doi: 10.1108/09578231011041062

[ref23] Darling-Hammond L. (2022). Reimagining American education: possible futures: the policy changes we need to get there. Phi Delta Kappan 103, 54–57. doi: 10.1177/00317217221100012

[ref24] Dawson G. (2008). Early behavioral intervention, brain plasticity, and the prevention of autism spectrum disorder. Dev. Psychopathol. 20, 775–803. doi: 10.1017/S0954579408000370, PMID: 18606031

[ref25] Day C. (2013). “The new lives of teachers” in Back to the future: Legacies, continuities and changes in educational policy, practice, and research. eds. Flores M. A. Carvalho A. A. Ferreira F. I. Vilaça M. T. (Dordrecht: Sense Publishers), 57–74.

[ref26] Day C. Kington A. Stobart G. Sammons P. (2006). The personal and professional selves of teachers: stable and unstable identities. Br. Educ. Res. J. 32, 601–616. doi: 10.1080/01411920600775316

[ref27] de Laat M. Schreurs B. (2013). Visualizing informal professional development networks: building a case for learning analytics in the workplace. Am. Behav. Sci. 57, 1421–1438. doi: 10.1177/0002764213479364

[ref28] Demir E. K. (2021). The role of social capital for teacher professional learning and student achievement: a systematic literature review. Educ. Res. Rev. 33:100391. doi: 10.1016/j.edurev.2021.100391

[ref31] DuFour R. (2003). Building a professional learning community. School Administrator 60, 13–18.

[ref32] Ferguson R. Buckingham S. (2012). Social learning analytics: five approaches. Proceedings of the 2nd international conference on learning analytics and knowledge, New York, NY

[ref33] Ferreira A. L. Brasil T. L. Acioly-Régnier N. M. (2018). O sociograma e os processos grupais: Uma experiência no campo educacional. Comunicações 25:137. doi: 10.15600/2238-121X/comunicacoes.v25n2p137-166

[ref34] Fetters M. D. (2016). “Haven’t we always been doing mixed methods research?”: lessons learned from the development of the horseless carriage. J. Mixed Methods Res. 10, 3–11. doi: 10.1177/1558689815620883

[ref35] Fournier T. Bruckert E. Czernichow S. Paulmyer A. Poulain J. P. (2011). The THEMA study: a sociodemographic survey of hypercholesterolaemic individuals. J. Hum. Nutr. Diet. 24, 572–581. doi: 10.1111/j.1365-277X.2011.01168.x, PMID: 21585569

[ref36] Gidari S. Kakana D. (2022). Using a mixed-method to evaluate a kindergarten teachers’ Professional development Programme and to investigate teachers’ Professional Growth

[ref37] Giddens A. (1999). Consecuencias de la modernidad.

[ref38] Glass C. R. Gesing P. (2018). The development of social capital through international students’ involvement in campus organizations. J. Int. Stud. 8, 1274–1292. doi: 10.5281/zenodo.1254580

[ref39] Grant R. M. (1996). Toward a knowledge-based theory of the firm. Strateg. Manag. J. 17, 109–122. doi: 10.1002/smj.4250171110

[ref41] Guskey T. R. (2002). Professional development and teacher change. Teachers and Teaching 8, 381–391. doi: 10.1080/135406002100000512

[ref42] Hammond M. (2019). A review of recent papers on online discussion in teaching and learning in higher education. Online Learn. 9:1782. doi: 10.24059/olj.v9i3.1782

[ref43] Hargreaves A. (2020). “The Day after: education and equity after the global pandemic” in Flip the system US: How teachers can transform education and save democracy. ed. Soskil M. (Boca Raton, FL: CRC Press), 64–73.

[ref44] Hargreaves A. Fullan M. (2012). Professional capital: Transforming teaching in every school. Ashland: Blackstone Publishing

[ref45] Hargreaves A. Fullan M. (2013). The power of professional capital. Learn. Profes. 34:36.

[ref46] Hargreaves A. Fullan M. (2020). Professional capital after the pandemic: revisiting and revising classic understandings of teachers' work. J. Prof. Capital Commun. 5, 327–336. doi: 10.1108/JPCC-06-2020-0039

[ref47] Hargreaves D. Miell D. Macdonald R. (2002). “What are musical identities, and why are they important” in Musical Identities (Oxford: Oxford University Press)

[ref48] Hargreaves A. O’Connor M. T. (2018). Collaborative professionalism: When teaching together means learning for all. 1st Edn. Thousand Oaks: Corwin.

[ref49] Harris D. N. Sass T. R. (2011). Teacher training, teacher quality and student achievement. J. Public Econ. 95, 798–812. doi: 10.1016/j.jpubeco.2010.11.009

[ref50] Haythornthwaite C. (2011). Learning networks, crowds and communities. Proceedings of the 1st international conference on learning analytics and knowledge-LAK’11, Banff, Alberta.

[ref51] Hernández E. Navarro M. J. (2018). La participación en redes escolares locales para promover la mejora educativa, un estudio de caso. Profesorado, Revista de Currículum y Formación del Profesorado 22, 71–90. doi: 10.30827/profesorado.v22i2.7715

[ref53] Hu S. Torphy K. T. Opperman A. Jansen K. Lo Y.-J. (2018). What do teachers share within socialized knowledge communities: a case of Pinterest. J. Prof. Cap. Community 3, 97–122. doi: 10.1108/JPCC-11-2017-0025

[ref54] Jan S. K. Vlachopoulos P. Parsell M. (2019). Social network analysis and online learning communities in higher education: a systematic literature review. Online Learn. 23:1. doi: 10.24059/olj.v23i1.1398

[ref55] Järvelä S. Kirschner P. A. Panadero E. Malmberg J. Phielix C. Jaspers J.. (2015). Enhancing socially shared regulation in collaborative learning groups: designing for CSCL regulation tools. Educ. Technol. Res. Dev. 63, 125–142. doi: 10.1007/s11423-014-9358-1

[ref56] Kavanaugh A. L. Patterson S. J. (2001). The impact of community computer networks on social capital and community involvement. Am. Behav. Sci. 45, 496–509. doi: 10.1177/00027640121957312

[ref57] Keithlucas A. (1957). Using the Sociogram in teaching Houseparents. Child Welfare 36, 1–7.

[ref58] Kerrigan M. R. (2015). Social Capital in Data-Driven Community College Reform. Community Coll. J. Res. Pract. 39, 603–618. doi: 10.1080/10668926.2013.866061

[ref59] Khalil M. Ebner M. (2016). What is learning analytics about? A survey of different methods used in 2013–2015. arXiv. doi: 10.48550/arXiv.1606.02878

[ref60] Khousa E. A. Atif Y. (2018). Social network analysis to influence career development. J. Ambient. Intell. Humaniz. Comput. 9, 601–616. doi: 10.1007/s12652-017-0457-9

[ref61] Kimble C. Hildreth P. (2008). Communities of practice-Vol. 2: Creating learning environments for educators. Charlotte, NC: Information Age Publishing.

[ref62] Kitchenham B. Charters S. (2007). Guidelines for performing systematic literature reviews in software engineering. Technical report, EBSE technical report EBSE-2007-01.

[ref63] Koedinger K. R. Kim J. Jia J. Z. McLaughlin E. A. Bier N. L. (2015). Learning is not a spectator sport: Doing is better than watching for learning from a MOOC. Proceedings of the second (2015) ACM conference on learning @ scale, New York, NY.

[ref64] Kovanovic V. Joksimovic S. Gasevic D. Hatala M. (2014). What is the source of social capital? The association between social network position and social presence in communities of inquiry. CEUR Workshop Proc. 1183, 21–28.

[ref66] Lave J. (1988). Cognition in practice: Mind, mathematics and culture in everyday life. Cambridge: Cambridge University Press.

[ref68] Leana C. R. (2011). The missing link in school reform. Standford Innov. Rev. 34, 3–4.

[ref69] LeCun Y. Bengio Y. Hinton G. (2015). Deep learning. Nature 521, 436–444. doi: 10.1038/nature14539 26017442

[ref70] Leong L. W. Ibrahim O. (2015). Role of information system (IS), social networking technology (SNT) and WEB 2.0 for improving learning outcomes: a case of Malaysian universities. Procedia Soc. Behav. Sci. 211, 111–118. doi: 10.1016/j.sbspro.2015.11.017

[ref71] Liberati A. Altman D. G. Tetzlaff J. Mulrow C. Gøtzsche P. C. Ioannidis J. P. A.. (2009). The PRISMA statement for reporting systematic reviews and meta-analyses of studies that evaluate health care interventions: explanation and elaboration. PLoS Med. 6:e1000100. doi: 10.1371/journal.pmed.1000100, PMID: 19621070 PMC2707010

[ref72] Liu X. Chang J. Zhang L. (2020). Development and validation of a scale for teacher professional capital for ICT-enhanced teaching innovation. 2020 Ninth International Conference of Educational Innovation through Technology (EITT), Porto, Portugal.

[ref73] Llopis-Albert C. Rubio F. (2021). Application of learning analytics to improve higher education. Multidiscip. J. Educ. Soc. Technol. Sci. 8:2. doi: 10.4995/muse.2021.16287

[ref74] Long P. Siemens G. (2011). What is learning analytics. In Proceedings of the 1st international conference learning analytics and knowledge, LAK, Banff, Alberta.

[ref75] Loomis C. P. (1948). The Most frequently chosen Sociogram. Sociometry 11, 230–234. doi: 10.2307/2785112

[ref76] Lu Q. Lu Q. Huang J. Ge Y. Wen D. Chen B.. (2020). EgoVis: a visual analysis system for social networks based on egocentric research. Int. J. Cooperat. Informat. Syst. 29, 1930003–1930002. doi: 10.1142/S0218843019300031

[ref77] Macfadyen L. P. Dawson S. (2010). Mining LMS data to develop an “early warning system” for educators: a proof of concept. Comput. Educ. 54, 588–599. doi: 10.1016/j.compedu.2009.09.008

[ref78] McKenzie J. van Winkelen C. Grewal S. (2011). Developing organisational decision-making capability: a knowledge manager’s guide. J. Knowl. Manag. 15, 403–421. doi: 10.1108/13673271111137402

[ref79] Michos K. Lang C. Hernández-Leo D. Price-Dennis D. (2020). Involving teachers in learning analytics design: lessons learned from two case studies. Proceedings of the Tenth International Conference on Learning Analytics & Knowledge. New York, NY.

[ref80] Minga-Vallejo R.-E. Ramírez-Montoya M.-S. Rodríguez-Conde M.-J. (2021). Methods for the evaluation of social learning (2017-2021): systematic literature review. Ninth international conference on technological ecosystems for enhancing Multiculturality (TEEM’21), Barcelona, Spain.

[ref81] Misanchuk M. Anderson T. (2002). Building Community in an Online Learning Environment: Communication, cooperation and collaboration. Available at: https://www.semanticscholar.org/paper/Building-Community-in-an-Online-Learning-and-Misanchuk-Anderson/cd6eaa59b4bc4688352dcf00858cadbea1588577

[ref82] Moher D. Liberati A. Tetzlaff J. Altman D. G. (2009). Preferred reporting items for systematic reviews and meta-analyses: the PRISMA statement. BMJ 339:b2535. doi: 10.1136/bmj.b2535, PMID: 19622551 PMC2714657

[ref83] Monés A. M. Damoulis Y. D. Acquila-Natale E. Álvarez A. Rodríguez M. C. Pérez R. C.. (2020). Achievements and challenges in learning analytics in Spain: the view of SNOLA. RIED 23:2. doi: 10.5944/ried.23.2.26541

[ref84] Moolenaar N. M. Sleegers P. J. C. Daly A. J. (2012). Teaming up: linking collaboration networks, collective efficacy, and student achievement. Teach. Teach. Educ. 28, 251–262. doi: 10.1016/j.tate.2011.10.001

[ref85] Muenchen R. A. (2011). R for SAS and SPSS users second edition conclusion. En R for SAS and Spss users, 2nd Edn (pp. 647–661). Berlin: Springer.

[ref86] Muñoz-Merino P. J. Moreno-Marcos P. M. Rubio-Fernández A. Tsai Y.-S. Gašević D. Delgado Kloos C. (2022). A systematic analysis of learning analytics using multi-source data in the context of Spain. Behav. Inform. Technol. 42, 643–657. doi: 10.1080/0144929X.2022.2039767

[ref88] Nahapiet J. Ghoshal S. (1998). Social capital, intellectual capital, and the organizational advantage. Acad. Manag. Rev. 23, 242–266. doi: 10.2307/259373

[ref89] Newmann F. King M. B. Rigdon M. (1997). Accountability and school performance: implications from restructuring schools. Harv. Educ. Rev. 67, 41–75. doi: 10.17763/haer.67.1.14141916116656q6

[ref90] Nonaka I. Takeuchi H. (1995). The knowledge-creating company: How Japanese companies create the dynamics of innovation. Oxford: Oxford University Press.

[ref91] Oktavia T. Sujarwo S. (2020). Exploration of recommender generator system to support social learning platform of higher education institution. ICIC Exp. Lett. 14, 489–496. doi: 10.24507/icicel.14.05.489

[ref92] Oliveira e Sá S. de Castro P. A. (2020). “Characteristics of the pedagogical supervisor in context of a constructive and reflective supervision” in Computer supported qualitative research. eds. Costa A. P. Reis L. P. Moreira A. (New York: Springer International Publishing), 274–287.

[ref93] Organization for Economic Co-operation and Development. (2019). Education at a glance. Available at: https://www.oecd-ilibrary.org/education/education-at-a-glance-2019_f8d7880d-en

[ref94] Paraschiv I. C. Dascalu M. McNamara D. S. Trausan-Matu S. (2016). “Finding the needle in a haystack: who are the most central authors within a domain?” in Adaptive and adaptable learning. eds. Verbert K. Sharples M. Klobučar T. (New York: Springer International Publishing), 632–635.

[ref95] Paredes W. C. Chung K. S. K. (2012). Modelling learning & performance: a social networks perspective. Proceedings of the 2nd international conference on learning analytics and knowledge, Vancouver, British Columbia.

[ref96] Paton R. M. Fluck A. E. Scanlan J. D. (2018). Engagement and retention in VET MOOCs and online courses: a systematic review of literature from 2013 to 2017. Comput. Educ. 125, 191–201. doi: 10.1016/j.compedu.2018.06.013

[ref97] Pedroso J. E. P. Jr. Siason N. D. Tangco-Siason A. (2021). Principal’s leadership practices during the COVID 19 pandemic: an exploratory study. Int. J. Arts Human. Stud. 1, 76–87. doi: 10.32996/ijahs.2021.1.1.12

[ref98] Peeters M. J. Vaidya V. A. (2016). A mixed-methods analysis in assessing students’ professional development by applying an assessment for learning approach. Am. J. Pharm. Educ. 80:77. doi: 10.5688/ajpe80577, PMID: 27402980 PMC4937972

[ref99] Penuel W. R. Frank K. A. Sun M. Kim C. M. Singleton C. A. (2013). The organization as a filter of institutional diffusion. Teach. Coll. Rec. 115, 1–33. doi: 10.1177/016146811311500105

[ref100] Pham M. C. Derntl M. Cao Y. Klamma R. (2012). Learning analytics for learning blogospheres. Proceedings of the 11th international conference on Advances in Web-Based Learning, Berlin, Heidelberg.

[ref101] Pollock A. Berge E. (2018). How to do a systematic review. Int. J. Stroke 13, 138–156. doi: 10.1177/1747493017743796 29148960

[ref102] Poole M. S. van de Ven A. H. (1989). Using paradox to build management and organization theories. Acad. Manag. Rev. 14, 562–578. doi: 10.2307/258559

[ref103] Popham W. J. (1999). Modern educational measurement: Practical guidelines for educational leaders 3rd Edn. London: Pearson.

[ref104] Raaper R. Brown C. (2020). The Covid-19 pandemic and the dissolution of the university campus: implications for student support practice. J. Prof. Cap. Community 5, 343–349. doi: 10.1108/JPCC-06-2020-0032

[ref105] Ranieri M. Manca S. Fini A. (2012). Why (and how) do teachers engage in social networks? An exploratory study of professional use of Facebook and its implications for lifelong learning. Br. J. Educ. Technol. 43, 754–769. doi: 10.1111/j.1467-8535.2012.01356.x

[ref106] Rincón S. (2019). “Las redes escolares como entornos de aprendizaje para los líderes educativos” in Cómo cultivar el liderazgo educativo. Trece miradas. eds. Weinstein J. Muñoz G. (Santiago: Universidad Diego Portales), 355–388.

[ref107] Rincón-Flores E. G. Montoya M. S. R. Mena J. (2019). Engaging MOOC through gamification: systematic mapping review. Proceedings of the Seventh International Conference on Technological Ecosystems for Enhancing Multiculturality, New York, NY.

[ref108] Rocco S. (2010). Making reflection public: using interactive online discussion board to enhance student learning. Reflective Pract. 11, 307–317. doi: 10.1080/14623943.2010.487374

[ref109] Ross J. Bruce C. (2007). Professional development effects on teacher efficacy: results of randomized field trial. J. Educ. Res. 101, 50–60. doi: 10.3200/JOER.101.1.50-60

[ref110] Salimi G. Heidari E. Mehrvarz M. Safavi A. A. (2022). Impact of online social capital on academic performance: exploring the mediating role of online knowledge sharing. Educ. Inf. Technol. 27, 6599–6620. doi: 10.1007/s10639-021-10881-w PMC8771188 35075344

[ref111] Sam Liu C.-H. (2017). Remodelling progress in tourism and hospitality students’ creativity through social capital and transformational leadership. J. Hospital. Leisure Sport Tourism Educ. 21, 69–82. doi: 10.1016/j.jhlste.2017.08.003

[ref112] Saunders R. (2014). Effectiveness of research-based teacher professional development. Australian J. Teach. Educ. 39, 5–6. doi: 10.14221/ajte.2014v39n4.10

[ref113] Schön E.-M. Thomaschewski J. Escalona M. J. (2017). Agile requirements engineering: a systematic literature review. Comput. Stand. Interfaces 49, 79–91. doi: 10.1016/j.csi.2016.08.011

[ref001] Schultz T. W. (1961). Investment in Human Capital. Am Econ Rev. 51, 1–17.

[ref114] Shepard S. Boudet H. Zanocco C. M. Cramer L. A. Tilt B. (2018). Community climate change beliefs, awareness, and actions in the wake of the September 2013 flooding in Boulder County, Colorado. J. Environ. Stud. Sci. 8, 312–325. doi: 10.1007/s13412-018-0479-4

[ref115] Siemens G. (2013). Learning analytics: the emergence of a discipline. Am. Behav. Sci. 57, 1380–1400. doi: 10.1177/0002764213498851

[ref116] Silva L. Mendes A. J. Gomes A. (2020). Computer-supported collaborative learning in programming education: a systematic literature review. 2020 IEEE Global Engineering Education Conference (EDUCON), Porto, Portugal.

[ref117] Simon H. A. (1977). The new science of management decision.

[ref118] Šmite D. Moe N. B. Šāblis A. Wohlin C. (2017). Software teams and their knowledge networks in large-scale software development. Inf. Softw. Technol. 86, 71–86. doi: 10.1016/j.infsof.2017.01.003

[ref119] Smylie M. A. Evans A. E. (2006). “Social capital and the problem of implementation” in New directions in education policy: Confronting complexity (Albany, NY: State University of New York Press), 187–208.

[ref120] Solórzano-García M. Navío-Marco J. (2019). Developing social entrepreneurs through distance education: the value of commitment and interactivity with the learning community. Int. J. Mobile Learn. Organ. 13, 30–50. doi: 10.1504/IJMLO.2019.096466

[ref121] Spillane J. P. Hopkins M. Sweet T. M. (2015). Intra-and interschool interactions about instruction: exploring the conditions for social capital development. Am. J. Educ. 122, 71–110. doi: 10.1086/683292

[ref122] Stoll B. J. Hansen N. I. Bell E. F. Shankaran S. Laptook A. R. Walsh M. C.. (2010). Neonatal outcomes of extremely preterm infants from the NICHD neonatal research network. Pediatrics 126, 443–456. doi: 10.1542/peds.2009-2959 20732945 PMC2982806

[ref123] Subekti A. Ferdiana R. Santosa P. I. (2019). Social media mapping for business communication. 2019 international conference on information and communications technology, ICOIACT 2019, Yogyakarta, Indonesia.

[ref124] Tahmasebi A. Askaribezayeh F. (2021). Microfinance and social capital formation-a social network analysis approach. Socio Econ. Plan. Sci. 76:100978. doi: 10.1016/j.seps.2020.100978

[ref125] Tatum P. E. Bell C. Hosokawa M. (2013). Use of a Sociogram to teach teamwork. J. Am. Geriatr. Soc. 61, S153–S154.

[ref127] Tong W. Razniak A. (2017). Building professional capital within a 21st century learning framework. J. Prof. Cap. Community 2, 36–49. doi: 10.1108/JPCC-06-2016-0018

[ref128] Tubaro P. Ryan L. D’angelo A. (2016). The visual Sociogram in qualitative and mixed-methods research. Sociol. Res. Online 21, 180–197. doi: 10.5153/sro.3864

[ref131] Verdu M. J. De Castro J.-P. Regueras L. M. Corell A. (2021). MSocial: practical integration of social learning analytics into Moodle. IEEE Access 9, 23705–23716. doi: 10.1109/ACCESS.2021.3056914

[ref132] Vieira C. Parsons P. Byrd V. (2018). Visual learning analytics of educational data: a systematic literature review and research agenda. Comput. Educ. 122, 119–135. doi: 10.1016/j.compedu.2018.03.018

[ref133] Visone J. D. (2018). Developing social and decisional capital in US National Blue Ribbon Schools. Improv. Sch. 21, 158–172. doi: 10.1177/1365480218755171

[ref134] Wang Z. Chen X. Yu M. (2017). Interactive effect of leader–member tie and network centrality on leadership effectiveness. Soc. Behav. Personal. Int. J. 45, 1197–1210. doi: 10.2224/sbp.6351

[ref135] Xing W. Gao F. (2018). Exploring the relationship between online discourse and commitment in twitter professional learning communities. Comput. Educ. 126, 388–398. doi: 10.1016/j.compedu.2018.08.010

[ref136] Yassine S. Kadry S. Sicilia M.-A. (2022). Detecting communities using social network analysis in online learning environments: systematic literature review. WIREs Data Mining Knowl. Discov. 12:e1431. doi: 10.1002/widm.1431

[ref138] Yoon S. A. Anderson C. W. Baker-Doyle K. de Laat M. de los Santos E. Frank K. A.. (2018). Networked by design: interventions for teachers to develop social capital. ICLS 2, 1235–1242.

[ref139] Yu X. Guan J. Leng J. (2016). Using learning analytics to support personalized learning and quality education: a case study of China’s “everyone connected” project. En Gong Z. Chiu D. K. W. Zou D. (Eds.), Current developments in web based learning (pp. 196–201). New York: Springer International Publishing.

[ref140] Zarra T. Chiheb R. Faizi R. El Afia A. (2018). MOOCs’ recommendation based on forum latent Dirichlet allocation. Proceedings of the 2nd international conference on smart digital environment, Rabat, Morocco.

[ref141] Zhang J. (2022). Teachers’ professional learning communities in China: a mixed-method study on Shanghai primary schools. London: Routledge.

[ref142] Zhao J. (2008). “A sociogram analysis on group interaction in an online discussion forum” in Advances in web based learning—Icwl 2008, proceedings. eds. Li F. Zhao J. Shih T. K. Lau R. Li Q. McLeod D., vol. 5145 (Berlin: Springer-Verlag), 377–389.

[ref143] Zhao J. (2009). Group interaction in a web 2.0 based learning environment: a sociogram analysis. Int. J. Contin. Eng. Educ. Life Long Learn. 19, 191–205. doi: 10.1504/IJCEELL.2009.025027

[ref144] Zhu M. Sari A. R. Lee M. M. (2022). Trends and issues in MOOC learning analytics empirical research: a systematic literature review (2011–2021). Educ. Inf. Technol. 27, 10135–10160. doi: 10.1007/s10639-022-11031-6

