# Supplementary material for: Learning analytics for enhanced professional capital development: a systematic review
Source: Front Psychol. 2024 Jan 22;15:1302658. doi: 10.3389/fpsyg.2024.1302658 (PMC10839147; doi:10.3389/fpsyg.2024.1302658)
Supplement: Supplementary file 1 [file Table_1.docx]

**ANNEXES.**

**ANNEX 1. Coding scheme used to summarize the data**

| **BIBLIOMETRIC DATA** | | | |
| --- | --- | --- | --- |
| **Year (Y)** | 2011, 2012, 2013, 2014, 2015, 2016, 2017, 2018, 2019, 2020, 2021 | | |
| **Repository (R)** | WOS (1) | | |
|  | SCOPUS (2) | | |
|  | ACM (3) | | |
| **Document types (DT)** | Articles (1) | | |
|  | Conference paper (2) | | |
|  | Book chapter (3) | | |
| **Journal / Editorial / Conference (JCT)** | CEUR Workshop Proceedings, American Behavioral Scientist, Higher Education (For more details about the coding of the rest of the journals, click on the link in the note of this table) | | |
| **Impact (I)** | Q1 JCR, Q2 JCR, , Q3 JCR, , Q4 JCR, ECR | | (JCR) Journal Citation Reports (Oviedo-García, 2021) |
|  | Q1 SJR, Q2 SJR, Q3 SJR, Q4 SJR | | (JRS) SCImago Journal Rank (Villaseñor-Almaraz et all, 2019) |
| **Country of authorship (C)** | 1. Canada 2. USA 3. Netherlands 4. England 5. Germany 6. Australia 7. Italy 8. Spain 9. Scotland 10. China 11. Russia 12. Ecuador 13. Singapore 14. Mexico 15. Sweden 16. Denmark 17. Israel 18. Brazil 19. Indonesia 20. Oman 21. Serbia 22. Finland 23. Malaysia 24. Saudi Arabia 25.Sri Lanka | | |
| **Source** | Conceptual (1) | | Framework developed through conceptual  research/theory |
|  | Empirical (2) | | Framework development is empirical |
|  | Mixed (3) | | Both of them |
| **Application Focus (AF) Stakeholders - who is the guidance for?** | Students (1.1.Kindergarten 1.2. Elementary school 1.3. High school 1.4. University students, 1.5. General) | | |
|  | Teachers (2.1.Kindergarten 2.2. Elementary school 2.3. High school 2.4 University Professors 2.5 General) | | |
|  | Students and teachers (3) | | |
| **TYPE OF PROFESSIONAL CAPITAL (TPC)** | | | |
| **Types of professional capital according to Hargreaves and Fullan (2014)** | 1. General Professional capital  2. Human capital  3. Decisional capital  4. Social capital | | |
| **DATA COLLECTION TYPE** | | | |
| **Collection medium (NR)** | 1. Virtual | | |
|  | 2. Face-to-face | | |
|  | 3. Blended | | |
| **Method of recollection (MNR)** | 1. Quantitative | | |
|  | 2. Qualitative | | |
|  | 3. Mixed | | |
| **Source (NS)** | 1. Surveys | (1. GD) Google drive / (1.D) Diary / (1.US) Unspecified survey | |
|  | 2. Digital platforms and services. (See Figure 1) | [2.1]-Blog and Wikis (2.1.B) Blogs (2.1.W) Wikis. | |
|  |  | [2.2.1 . ITS] - Intelligent Tutoring System (2.2.1.F) Forums (2.2.1.C) Chats (2.2.1. UW) Unspecified  [2.2.2.LMS]- Learning Management System. (2.2.2.F) Forums (2.2.2.C) Chats (2.2.2. UW) Unspecified  [2.2.3.] MOOCS, (2.2.3.F) Forums (2.2.3.C) Chats (2.2.3. UW) Unspecified | |
|  |  | [2.3.] Social Networks (2.3.FA) Facebook / (2.3.TW) Twitter (2.3. I) Instagram (2.3.LI) LinkedIn (2.3. UW) Unspecified ] | |
|  | 3. Interviews. | (3.SI) Semi-structured interview | |
| **DATA ANALYSIS TYPE** | | | |
| **Analysis medium (NA)** | 1. Virtual | | |
|  | 2. Face-to-face | | |
|  | 3. Blended | | |
| **Techniques used for analysis (TENA)** | 1. Data mining | (1.A) Algorithms | |
|  |  | (1.C) Clustering | |
|  |  | (1.U) Unspecified | |
|  | 2. Statistics | | |
|  | 3. Text mining, semantic and linguistic analysis | (3.CA) Content analysis | |
|  |  | (3.SA) Structural analysis | |
| **Tools used for analysis (TONA)** | 1. NICU, 2. Pajek, 3. Gephi, 4. NetDraw, 5. SPSS, 6- SNA software unspecified (For more details about the tools, click on the link in the note in this table) | | |
| **Visualization (V)** | 1. Sociograms, 2. dispersion diagram, 3. line graphs, 4. bubble charts (For more details about the visualization, click on the link in the note in this table) | | |
| **PROGRAMS TO IMPROVE PROFESSIONAL CAPITAL** | | | |
| **Improvement medium (PTN)** | 1. Virtual  2. Face-to-face  3. Blended | | |
| **Programs or Tools (FVB)** | 1. Openstudy, 2. The Network Awareness Tool (For more details about the programs, click on the link in the note in this table) | | |
| **Digital platforms and services (DPS)** (See Figure 1) | 1. Blog and wikis | | |
|  | 2. ITS- Intelligent Tutoring System  3. LMS-Learning Management System  4. MOOCS | | |
|  | 5. Social networks or social media. | | |
| **VARIABLES THAT INFLUENCE AND ARE INFLUENCED BY SOCIAL CAPITAL** | | | |
| **Variables that influence social capital (VSC)** | 1. Motivation, 2. Affection, 3. Trust , 4. Engagement, and 5. Content Richness (see the rest of the variables in the Results section) | | |
| **Variables influenced by social capital (SCV)** | 1. Learning, 2. Achievement, 3.Engagement. 4. Motivation, and 5. Performance (see the rest of the variables in the Results section) | | |
| **EMERGENT TOPICS (ET)** (Qiao et all., 2018; Baber et all., 2022) | | | |
| 1. Covid-19, 2. Sustainable development goals, 3. Informal Learning and 4. Collaborative learning | | | |

*Notes:* Full analysis at the following link (Open access):<https://drive.google.com/file/d/1PfJcaXrjcz9S3vmSpHlo42mbNegkmgtf/view?usp=share_link>
